# Sex-Specific Gene Expression Differences in Varicose Veins

**DOI:** 10.3390/biomedicines13102373

**Published:** 2025-09-27

**Authors:** Mariya A. Smetanina, Valeria A. Korolenya, Ksenia S. Sevostyanova, Konstantin A. Gavrilov, Fedor A. Sipin, Andrey I. Shevela, Maxim L. Filipenko

**Affiliations:** 1 Laboratory of Pharmacogenomics, Institute of Chemical Biology and Fundamental Medicine (ICBFM) SB RAS, Novosibirsk 630090, Russia; 2 Department of Fundamental Medicine, V. Zelman Institute for Medicine and Psychology, Novosibirsk State University (NSU), Novosibirsk 630090, Russia; 3 Department of Natural Sciences, Novosibirsk State University (NSU), Novosibirsk 630090, Russia; 4 Center of New Medical Technologies, Institute of Chemical Biology and Fundamental Medicine (ICBFM) SB RAS, Novosibirsk 630090, Russia; 5 Laboratory of Invasive Medical Technologies, Institute of Chemical Biology and Fundamental Medicine (ICBFM) SB RAS, Novosibirsk 630090, Russia; 6 Department of Surgical Diseases, V. Zelman Institute for Medicine and Psychology, Novosibirsk State University (NSU), Novosibirsk 630090, Russia

**Keywords:** varicose veins, chronic venous disease, sex differences, gene expression

## Abstract

**Background/Objectives**: There is clear evidence for the higher prevalence of varicose veins (VVs) among women. In this regard, the research on sex differences affecting this condition is very important for sex-specific health care. We aimed to assess how male or female sex may contribute to the changes to gene expression profiles in the vein wall during varicose transformation. **Methods**: Paired varicose vein (VV) and non-varicose vein (NV) segments were harvested from patients with VVs after venous surgery. Processed RNAs from those samples were subjected to gene expression analysis by reverse transcription quantitative polymerase chain reaction (RT-qPCR) followed by further data analysis. Multiple linear regression (MLR) analysis was performed to identify and characterize relationships among multiple factors (relative mRNA levels of a gene in NV or VV or their ratio, as dependent variables) and sex (independent variable, used individually or in combination with other patient’s characteristics). For sex-specific gene regulation analysis, all potential binding sites for sex hormone receptors were identified in each gene’s regulatory region sequence. **Results**: Using the independent method and a replicative patient sample set, we validated our previous data on 23 genes’ differential expression in VVs and obtained insights on their sex-specific regulation. Sex (as an individual independent variable or in combination with other parameters—patient characteristics such as Age, BMI, CEAP class, Height, VVD manifestation and duration) was a moderate predictor (0.40 < R < 0.59; *p* (R) < 0.05) for the *STK38L* expression in VVs (with its higher mRNA level in NVs and VVs of women compared to men); sex was a strong predictor (0.6 < R < 0.79; *p* (R) < 0.05) for the *TIMP1* expression in VVs (with its lower mRNA level in VVs of women compared to men); sex was a moderate predictor (0.40 < R < 0.59; *p* (R) < 0.05) for the *EBF1* expression in NVs (with its lower mRNA level in NVs of women compared to men). **Conclusions**: Confirmed differential expression of the studied genes in VVs indicates their plausible participation in vein wall remodeling. Sex-specific expression in veins for the subset of those genes suggests their hormonal regulation as well as other mechanisms involved in VV pathogenesis. This work enriches our understanding of sex features for the development of VVs and may provide the foundation for future investigations and beneficial treatment options.

## 1. Introduction

Varicose veins (VVs) are the most common form of chronic venous disease [1], defined as dilated, tortuous superficial veins with deformation of the vessel wall accompanied by valve insufficiency and impaired blood flow [2]. This condition corresponds to class C2 according to the clinical, etiological, anatomical, and pathophysiological (CEAP) classification [3], the global prevalence of which is 19% [1]. The absence of timely treatment of VVs leads to complications such as edema and venous ulcers, so the presence of VVs defines a widespread condition with serious consequences for the patient’s life [2].

It is generally accepted that females have a higher risk factor in the development of VVs [4], which is confirmed by a number of studies [5,6,7,8,9]. While the estimate of this prevalence varies greatly, this may indicate different approaches to the assessment of VVs and different geographic, political, national, and mentality features of the region in which the assessment was carried out. According to 28 studies, the frequency of VVs was estimated as 2 to 56% in men and <1 to 73% in women by Beebe-Dimmer et al. [4]. Nevertheless, there are studies that refute the predominance of VVs in women [10,11,12].

The reasons for the higher prevalence of VVs among women may be genetic, physiological, and anatomical features. Helkkula et al. examined the evidence for sex differences in VV genetics in the Finnish population and, in spite of high similarity in genetic effects and variance between men and women, identified that one female-specific locus near the *ERG* gene located on the 21 chromosome and two loci located on the X chromosome were associated with VV risk [9]. Due to the presence of a second X chromosome, women have a more active immune system [13], which may contribute to the pathogenesis of VVs characterized by inflammation [14]. Hormonal background changes make a major contribution to the pathogenesis of VVs. Strong arguments in favor of the higher prevalence of VVs among women include specific risk factors like pregnancy, childbirth, and menopause along with hormone replacement therapy, wearing high heels, and taking oral contraceptives. In a meta-analysis by Ismai et al., it was shown that a history of pregnancy increases the risk of VVs by 82% [15]. Mullane found a dependence of the incidence of VVs on the number of childbirths; the study showed that 13% of primiparous, 30% of secundiparous, and 57% of multiparous women suffer from VVs [16]. Such values may be a consequence of physiological changes associated with pregnancy and childbirth [17,18] and additional stress on the mother’s cardiovascular system [17]. A decrease in estrogen levels in women during menopause also contributes to deformation of the venous wall, which is manifested by a 2-fold increase in the risk of VVs [12]. Differences in skeleton, muscle, and adipose tissue distribution may also contribute significantly to this pathology. A study by Baldazzi et al. showed that the venous reflux related to VVs differs between the sexes: they found a predisposition to an ascending modality of VV progression in women (due to insufficient tributaries), while a descending mechanism is suggested in men (due to incompetence of the saphenofemoral junction) [19].

Epigenetic features defined by sex are of great importance in the development of cardiovascular diseases, which emphasizes the need to take into account sex characteristics when prescribing therapy [20]. Sex may influence gene expression, particularly in the venous wall, which can govern the course of pathogenesis and susceptibility to treatment. García-Honduvilla et al. showed differences in the expression of sex hormone receptors between the sexes (in norm and pathology) [21]. Unfortunately, there are no other data from the available literature on the difference in gene expression in healthy and varicose veins between men and women. We have previously published studies dedicated to changes in gene expression in the venous wall upon varicose transformation [22,23,24,25,26,27,28,29], some of them only as abstracts in conference proceedings [30,31,32,33,34,35]; however, we did not test the influence of sex on its relative values. Based on the above, in the present study we used some new data, as well as our previous data, to conduct an additional analysis to identify the effect of sex on the expression of genes involved in varicose transformation. Investigation of sex distinctions in the pathogenesis and treatment susceptibility of chronic venous diseases will help to provide a more personalized approach to the diagnosis and treatment of these conditions.

## 2. Materials and Methods

### 2.1. Participants of the Study

**Figure 1:**
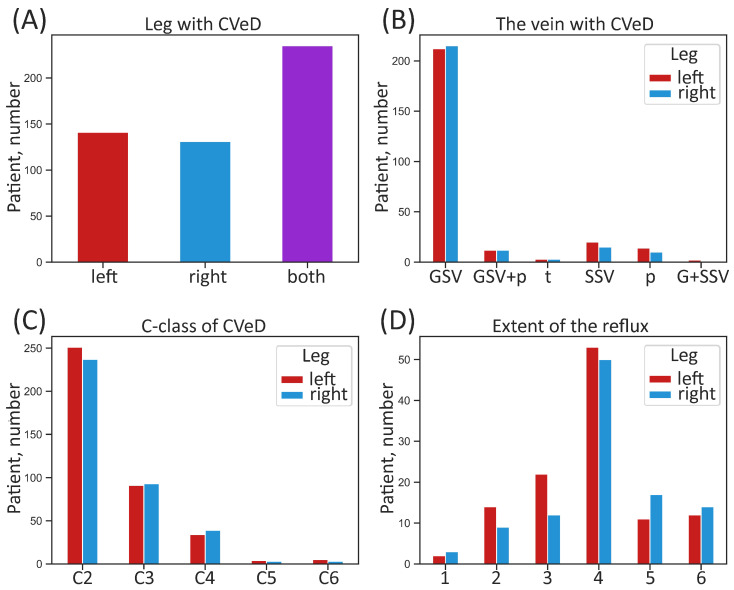
Categorical characteristics of the whole sample of patients according to leg. (**A**) Number of patients suffering from CVeD in the right, left, or both legs; (**B**) number of patients with different anatomical manifestation of CVeD; (**C**) number of patients with different clinical class of CVeD; (**D**) number of patients with different extent of pathological venous reflux. CVeD—chronic venous disease; GSV—great saphenous vein; G + SSV—small and great saphenous vein; GSV + p—great saphenous vein and perforators; SSV—small saphenous vein; p—perforators; t—tributaries; C-class (C)—clinical class according to the CEAP classification. The extent of pathological venous reflux is coded as follows: 1—to the upper third of the thigh, 2—to the middle third of the thigh, 3—to the lower third of the thigh, 4—to the upper third of the calf, 5—to the middle third of the calf, 6—to the lower third of the calf.

Patients (162 men and 368 women, mean age 47 years) had a clinical diagnosis of mostly C2–C4 (i.e., VVs requiring medical intervention) according to the CEAP classification. Exclusion criteria were absence of visible VVs and post-thrombotic changes in deep veins on the leg with VVs. Descriptive (categorical) characteristics of the patient sample studied according to leg are given in Figure 1.

**Figure 2:**
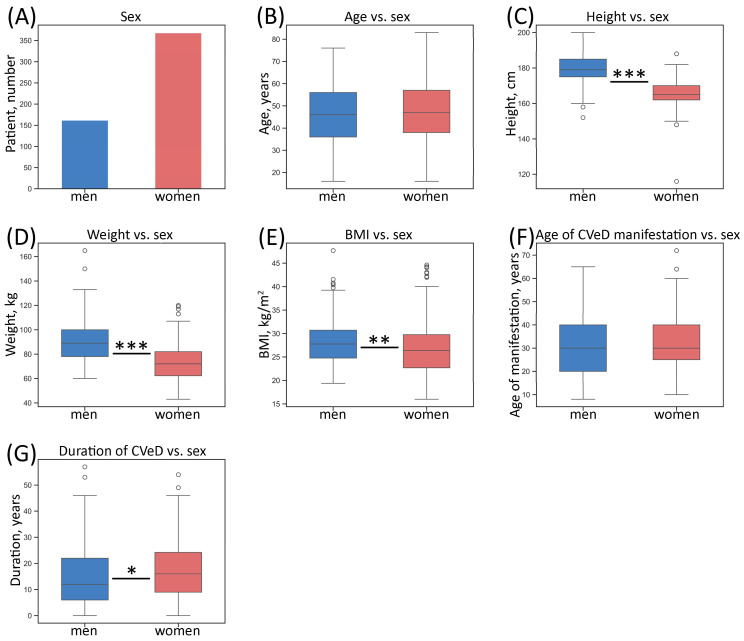
Numerical characteristics of the whole sample of patients according to sex. (**A**) Number of patients; (**B**) distribution of age; (**C**) distribution of height; (**D**) distribution of weight; (**E**) distribution of BMI; (**F**) distribution of age of CVeD manifestation; (**G**) distribution of duration of CVeD. The box borders show the interquartile range (IQR), the horizontal line inside it indicates the median, and the whiskers show the 1.5 of IQR, points outside the whiskers represent numerical outliers (>1.5 of IQR); * *p*-value < 0.05, ** *p*-value < 0.01, *** *p*-value < 0.001 (Mann–Whitney U test); BMI—body mass index.

Instead of tables, figures describing several parameters of the study cohort were generated for better visualization and perception. Descriptive (numerical) characteristics of the studied patient sample according to sex are given in Figure 2.

The entire study sample consisted of 530 individuals with VVs who underwent varicose vein surgery in 2013–2021. Numbers of patients in the sample subsets intended to evaluate the expression of one particular gene varied from 12 to 35 corresponding to 24–70 paired varicose vein (VV) and non-varicose vein (NV) segments.

### 2.2. Sample Collection, RNA Extraction, and Processing

Paired VV and NV segments (case and control, respectively) of the basin of the great saphenous vein (GSV) were harvested from each patient with VVs. Surgical material was immediately placed in liquid nitrogen (for snap-freezing) then put at –80 °C until the time to be used. Total RNAs were isolated from homogenized vein samples according to the protocol used by Korolenya et al. [36], and their integrity was confirmed by visualization of intact 18S and 28S rRNA under UV light. For equal conditions in subsequent qPCR reactions, RNA samples were made equal in concentrations though further normalization to reference genes in each sample was performed during the data analysis. Then, the cDNAs were synthesized using the reverse transcriptase (DNA-Synthesis, Moscow, Russia) in accordance with the instructions given by Korolenya et al. [36]. Synthesized cDNA samples were diluted 10-fold with nuclease-free water and taken for gene expression analysis.

### 2.3. Gene Expression Analysis by Reverse Transcription qPCR

Determination of relative mRNA level (normalized to reference genes) was performed by SYBR Green-based qPCR using a CFX-96 thermal cycler (Bio-Rad, Hercules, CA, USA). Primer design was performed using Oligo Analyzer (version 1.0.0) and Annhyb (version 2.22) software. Primer sequences are given in Supplementary Table S1. A subset of reference genes—*ACTB* and *GAPDH*—was selected to be used for normalization according to our geNorm-analysis using qBase+ software (version 3.2) carried out earlier (*ACTB*, *GAPDH*, and *POLR2A* were the 3 most stable housekeeping genes expressed in venous samples) [22].

Amplification mixtures (20 µL) contained up to 25 ng of template cDNAs (calculated per RNAs), forward and reverse primers (300 nM), Taq polymerase (2 U) per reaction (DNA-Synthesis), and buffer (10 mM Tris-HCl (pH 8.9), 25 mM MgCl2, 55 mM KCl, 0.2 mM dNTPs, and 0.1% Tween-20). SYBR Green I was used as an intercalating dye. The following PCR program was used: 96 °C for 3 min, then 40 cycles of 96 °C for 10 s, 58–62 °C for 10 s, 72 °C for 10 s, and 75–87 °C for 5 s (for collecting fluorescent signals at SYBR channel). Each measurement was performed in technical triplicate and included a standard curve of four serial dilution points of mixed samples’ cDNA, a no-template control, and each test cDNA. For quantifying small differences in relative gene expression, we used the standard curve method as more reliable compared to the ΔΔCt method, since PCR efficiency for one primer pair may fluctuate from run to run. An outlier replicate was excluded from the analysis for the samples with standard deviation of ΔCq values of the three replicates > 0.5. For all qPCR systems, standard curves were generated using the same reference sample prepared from the vein according to the same protocol as for all the samples analyzed. Bio-Rad CFX Maestro results were exported as Excel and Rdml files and imported into qBase+ software (version 3.2) for further analysis according to the software manual and additional literature [37,38].

### 2.4. Statistical Analysis

Statistical analysis was performed using qBase+ (version 3.2), Microsoft Excel (version 2508, build 19127.20240), STATISTICA (version 8.0), and Past (version 4.16c) software. All the graphs were generated using data visualization libraries for Python (version 3.9.10)—Matplotlib (version 3.7.1) and Seaborn (version 0.13.2). Box plots were constructed to visualize the distribution of values: the horizontal line inside the box indicates the median, the box borders show the interquartile range (IQR)—values between the 1st and 3rd quartiles—and the whiskers show the maximum and minimum values. In the case of describing numerical characteristics of the patient sample (Figure 2), the whiskers show the 1.5 of IQR, and points outside the whiskers represent numerical outliers (>1.5 of IQR).

Sample data distribution was checked using the Shapiro–Wilk, Kolmogorov–Smirnov, and Lilliefors normality tests. Differences between men and women were tested using Student’s *t*-test or Mann–Whitney U test, depending on the normality. Gene expression differences between paired VV and NV segments were analyzed mostly by the one-sample Wilcoxon signed-rank test (when the distribution of gene expression relative values was not normal); in case of normal distribution (only for 3 genes), paired Student’s *t*-test was applied. To detect associations between sex and another categorical feature, the chi-square test was used (results are shown in heatmaps). Statistical significance threshold for all the tests was set at *p*-value < 0.05.

### 2.5. Multiple Linear Regression Analysis

Multiple linear regression (MLR) analysis was performed using STATISTICA (version 8.0), Past (version 4.16c), and Microsoft Excel (version 2508, build 19127.20240) software. MLR theoretically could enable us to (a) describe relationships among the independent variable sex and the dependent variables (relative mRNA levels), (b) estimate the values of the dependent variables from the observed independent variable sex, and (c) identify risk factors which influence the outcome and determine individual prognoses [39]. For correct variables combination and procedures, we followed the recommendations described by Schneider et al. [39]. To avoid excessive collinearity and confounding, ‘BMI’ (instead of ‘Weight’) was left in the independent variable list since it reflects weight adjusted to height and is also not collinear to it. ‘Forward’ method was selected for a stepwise or ridge regression procedure. We had to exclude some samples from MLR analysis in cases where some of the questionnaires with patient characteristics missed a few data. To ensure regression model stability, we performed the multicollinearity diagnostics [40]. Namely, variance inflation factor (VIF) analysis was carried out for simple linear regression when sex was the only independent predictor variable^#^ (being nominal categorical—binary variable containing only two mutually exclusive categories—men or women). Generalized variance inflation factor (GVIF, computed using the corresponding VIF values) analysis was carried out for multiple linear regression when all parameters (sex and other independent predictor variables including non-binary categorical variable ‘CEAP class’) were applied together*. The formulas were VIF = 1/(1 − R^2^) and GVIF = VIF^(1/(2×*df)), where R^2^ is a coefficient of multiple determination, and df is a degree of freedom. The results are shown in the last column of Supplementary Table S2. We considered that multicollinearity is present when the VIF was higher than 5 to 10 (average threshold used is often 4 to 5), and GVIF values above 2.5 to 5 (average threshold used is often 3.16). The residual analysis (accessing Durbin–Watson d and serial correlation of residuals) in STATISTICA (version 8.0) software was additionally performed to reveal instability linked to uneven sample sizes. For labeling the strength of the association, the following criteria were used for absolute values of r/R: 0–0.19 was considered as very weak correlation, 0.2–0.39 as weak correlation, 0.40–0.59 as moderate correlation, 0.6–0.79 as strong correlation, and 0.8–1 as very strong correlation [41].

When performing MLR analysis ‘Sex predictor variable + all other parameters vs. gene expression relative values’, independent predictor variables (patient characteristics such as sex, age, BMI, VVD manifestation, CEAP class, height, and VVD duration, available to us from the questionnaires) were applied together in order to observe their possible effects on each of the dependent variables: ‘gene expression relative value in NV’, ‘gene expression relative value in VV’, or ‘NV:VV or VV:NV ratio of gene expression relative values’.

When performing MLR analysis ‘Sex predictor variable separately vs. gene expression relative values’ (analog of simple linear regression) we used all three variables (‘gene expression relative value in NV’, ‘gene expression relative value in VV’, and ‘NV:VV or VV:NV ratio of gene expression relative values’) in order to visualize the results in one graph since there was no confounding between them. The graphs with scatter plots and corresponding regression lines for the relationship between the dependent (gene expression) variable and the independent (sex) variable were (a) incorporated in the corresponding figures within the Section 3 for the genes with certain significance or (b) presented in Supplementary Figures S1–S20 for the genes with incomplete significance.

Brief summary tables of the sex-related MLR analysis results for gene expression that showed certain significance were incorporated in the corresponding figures in the Section 3. Full summary table of the sex-related MLR analysis results on the expression of genes with incomplete significance (as well as of those genes described in the Section 3 is presented as Supplementary Table S2.

### 2.6. Sex-Specific Gene Regulation Analysis Through Identification of Sex Hormone-Related Transcription Factor Binding Sites

In order to obtain insights on the sex-specific regulation of the studied genes, a search for sex hormone-related transcription factor binding sites (TFBS) within the regulatory regions of these genes containing their promoters and associated enhancers and silencers was carried out using the MATCH Suite (release 3.1) software integrated into the TRANSFAC^®^ 2.0 solution for gene regulation analysis at https://genexplain.com/transfac (accessed on 26 July 2025) [42]. The focus was set up for TFBS for transcription factors acting themselves as nuclear receptors for sex hormones as their ligands; therefore, TFBS corresponding to androgen receptors (AR), estrogen receptors (ER), and progesterone receptors (PR) were considered in particular. Whole analysis workflow was performed according to the manufacturer’s MATCH Suite User guide release 3.1 (geneXplain). We analyzed the most active promoters in the range of [−1000, +100] bp from TSS and all known enhancers and silencers associated with those genes. Only matrices associated with transcription factors belonging to the ‘Reproductive system development’ Gene Ontology category <GO:0061458> were taken into the constructed profile. The selection of the most promising transcription factors regulating the input genes was based on complex criteria including (1) estimation of cumulative binding affinity of the transcription factors to the analyzed regulatory regions, and (2) high average expression of these factors among all tissues and their expression specificity values. In the regulatory regions of the input genes, potential TFBS were identified and checked for cumulative binding affinity. Using the MATCH™ algorithm, all potential binding sites for sex hormone receptors were identified in each regulatory region sequence. The data were extracted in respective tables for further analysis.

## 3. Results

### 3.1. The Associations Between Sex and Some Descriptive Characteristics of Patients

**Figure A1:**
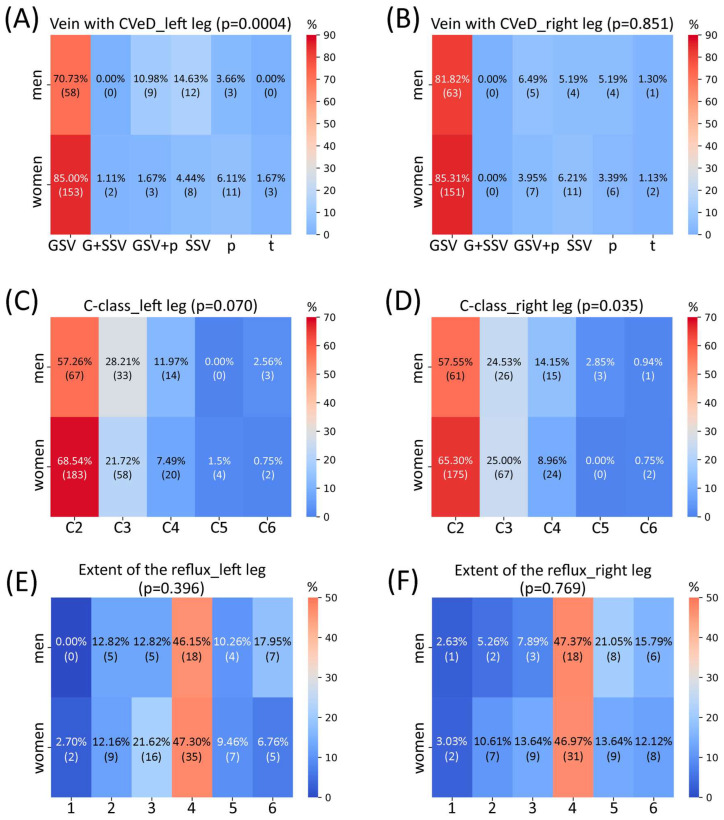
Heatmaps depicting the percentage of patients (among those patients who provided data in the questionnaires) and their number in the brackets on descriptive characteristics (categorical parameters) according to sex. (**1st Panel**): percentage of patients with different anatomical manifestation of CVeD in the left (**A**) and right (**B**) leg; (**2nd Panel**): percentage of patients with different clinical class of CVeD in the left (**C**) and right (**D**) leg; (**3rd Panel**): percentage of patients with a different extent of pathological venous reflux in the left (**E**) and right (**F**) leg. Colors represent the percentage levels from low (blue) to high (red); *p*-value (in the brackets) shows the likelihood of the data occurring under the null hypothesis of the chi2 test (categorical parameter vs. sex). CVeD—chronic venous disease; GSV—great saphenous vein; G + SSV—small and great saphenous vein; GSV + p—great saphenous vein and perforators; SSV—small saphenous vein; p—perforators; t—tributaries; C-class (C)—clinical class according to the CEAP classification. The extent of pathological venous reflux is coded as follows: 1—to the upper third of the thigh, 2—to the middle third of the thigh, 3—to the lower third of the thigh, 4—to the upper third of the calf, 5—to the middle third of the calf, 6—to the lower third of the calf.

Anterior to evaluation of sex-related differences in the gene expression in varicose veins, we decided to assess possible connections between sex of the patients and their descriptive characteristics according to the information provided in the questionnaires. To detect associations between sex and another categorical parameter, we used the chi-square test. The results were generated as heatmaps and presented in Appendix A. According to our population-specific data, there are statistically significant differences between men and women in anatomical manifestation of CVeD in the left leg (Figure A1A) and clinical class of CVeD in the right leg (with a tendency for the left leg) (Figure A1C,D). There is a prominent difference between men and women in CVeD manifestation in great saphenous vein + perforators (10.98% in men vs. 1.67% in women), as well as in small saphenous vein only (14.63% in men vs. 4.44% in women) of the left leg, albeit women are more likely to develop CVeD only in the great saphenous vein of the left leg compared to men (85% vs. 70.73%, correspondingly). One can see that men tend to develop CVeD of a more severe clinical class (C4–C6). There was no difference in the extent of pathological venous reflux between men and women.

**Figure A2:**
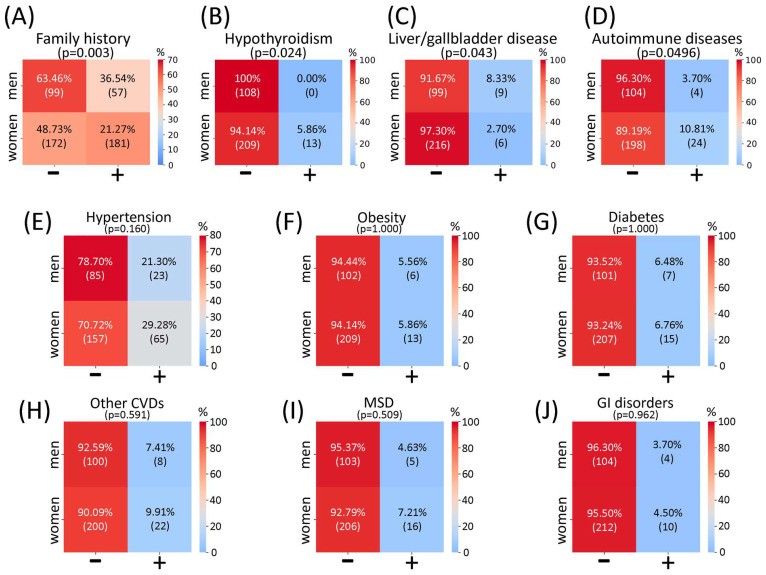
Heatmaps depicting the percentage of patients (among those patients who provided data in the questionnaires) and their number in the brackets on family history for CVeD and comorbidities (categorical parameters) according to sex. Percentage of patients without (−) and with (+) family history for CVeD (**A**), and patients not suffering (−) and suffering (+) from hypothyroidism (**B**), liver and/or gallbladder diseases (**C**), autoimmune diseases (**D**), hypertension (**E**), obesity (**F**), diabetes (**G**), other cardiovascular diseases (**H**), musculoskeletal disorders (**I**), and gastrointestinal disorders (**J**). Colors represent the percentage levels from low (blue) to high (red); *p*-value (in the brackets) shows the likelihood of the data occurring under the null hypothesis of the chi2 test (categorical parameter vs. sex). CVeD—chronic venous disease; CVDs—cardiovascular diseases; MSDs—musculoskeletal disorders; GI—gastrointestinal.

Upon assessment of possible associations between sex and such categorical parameters as family history for CVeD and comorbidities, it was shown that compared to women, a larger percentage of men had CVeD family history (36.54% in men vs. 21.27% in women) and liver and/or gallbladder diseases (8.33% in men vs. 2.7% in women), albeit a larger percentage of women, compared to men, suffered from hypothyroidism (5.86% in women vs. 0% in men) and autoimmune diseases (10.81% in women vs. 3.7% in men) (Figure A2A–D).

### 3.2. Gene Expression Analysis Reveals a Set of Genes with Altered mRNA Level in VVs

In this study, we first aimed to validate, using an independent method and a replicative patient sample set, our microarray data [22] on a set (23) of genes differentially expressed in varicose venous segments compared to normal ones in patients with varicose veins. Using reverse transcription quantitative polymerase chain reaction (RT-qPCR) we verified altered expression for a set of genes listed in Table 1. For three of those genes (*CHRDL2*, *COMP*, and *MFAP5*) we reconfirmed their validated differential expression on a larger sample set.

The illustration of the Table data (distribution of gene expression relative values in NV vs. VV segments in the whole patient sample) is presented in Figure 3A, Figure 4A and Figure 5A (shown further) and Supplementary Figures S1A–S20A. Using an independent method, we revealed that 16 genes (*CALU*, *CASZ1*, *CCL2*, *CHRDL2*, *COL15A1*, *COMP*, *EFEMP1*, *GPI*, *ITGA5*, *MFAP5*, *MYO18B*, *PLA2G2A*, *STK38L*, *SULF1*, *TIMP1*, *TNC*) are upregulated (↑) and 7 genes (*ABCA1*, *AXL*, *EBF1*, *Mn-SOD*, *MYOD1*, *PPP1R12B*, *VCL*) are downregulated (↓) in VVs. These findings are consistent with our previous transcriptome analysis. Changes in the level of transcripts of these genes in VVs indicate their plausible participation in the remodeling of the venous wall.

### 3.3. The Associations Between Sex and Differential Expression of Some Genes in Patients’ Veins

Then, we evaluated sex-related differences in the expression of the genes differentially expressed in VVs validated by RT-qPCR (Table 1). For this, we measured gene expression differences (in mRNA relative values) regarding an independent predictor variable—sex. Initially, we searched for possible differences in gene expression between men and women (a) within NV samples and (b) within VV samples, as well as (c) within NV/VV or VV/NV paired samples’ ratio, depending on the downregulation or upregulation of a gene, which could also be informative, for each gene from our list. It turned out, that only in case of three genes—the *STK38L*, *TIMP1*, and *EBF1*—certain differences were observed (see Figure 3B, Figure 4B and Figure 5B in the text below) compared to other genes investigated (see Supplementary Figures S1B–S20B). Therefore, we decided to illustrate the results for these genes within the main text.

To see whether there is a relationship between each dependent variable—normalized gene expression value that reflects relative mRNA level of a gene in NV or VV or their ratio (NV/VV or VV/NV, depending on the downregulation or upregulation of a gene) and the independent variable—and sex, scatter diagrams for all genes were constructed. The results are shown in the graphs with correlations (scatter plot and the corresponding regression line) in Figure 3C, Figure 4C and Figure 5C in the text below for the *STK38L*, *TIMP1*, and *EBF1* genes or in Supplementary Figures S1C–S20C for other genes investigated.

**Figure 3:**
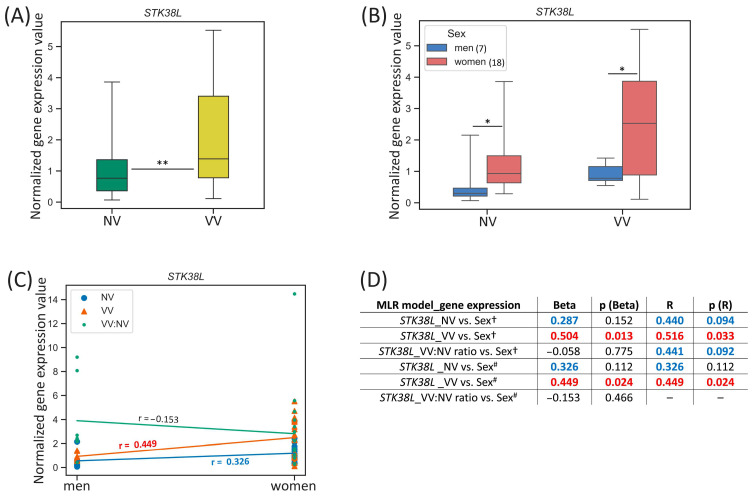
*STK38L* gene expression (mRNA level) data analysis. (**A**) Distribution of gene expression relative values in paired non-varicose vs. varicose vein segments in patients of the whole sample; (**B**) distribution of gene expression relative values in non-varicose and varicose vein segments according to sex; (**C**) scatter plot and the corresponding regression line for the relationship between the dependent (gene expression) variable and independent (sex) variable; (**D**) multiple linear regression analysis results of the *STK38L* gene expression. The box borders show the interquartile range, the horizontal line inside it indicates the median, and the whiskers show the maximum and minimum values; NV—non-varicose vein; VV—varicose vein; MLR—multiple linear regression; ** *p*-value < 0.01, * *p*-value < 0.05 (Wilcoxon test (**A**), Student’s *t*-test, Mann–Whitney U test (**B**)); ^†^ all parameters (independent predictor variables) applied together: sex, age, BMI, VVD manifestation, CEAP class, height, VVD duration (data are provided only for sex); ^#^ sex is the only independent predictor variable; Beta—regression coefficient; R—coefficient of multiple correlation (the positive square root of the coefficient of multiple determination); *p* (Beta)—*p*-value of Beta; *p* (R)—*p*-value of R; Beta and R > |±0.25| are displayed in blue at *p*-value > 0.05; Beta and R > |±0.25| are displayed in red at *p*-value < 0.05; 0.05 < *p*-values < 0.1 are displayed in blue; *p*-values < 0.05 are displayed in red; r—correlation coefficient; r > |±0.3| is displayed in blue; r > |±0.5| is displayed in red; the number of patients of different sexes is indicated in the legend in the brackets; empty graphs (“–”) mean that MLR analysis results are not available (there were no variables in the regression equation).

**Figure 4:**
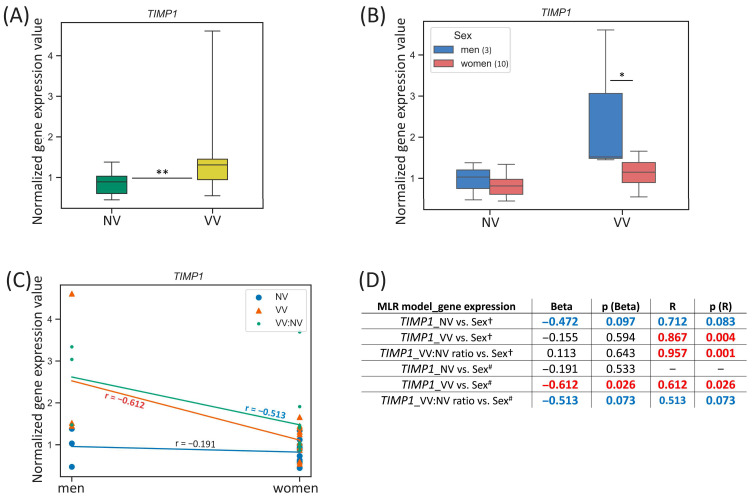
*TIMP1* gene expression (mRNA level) data analysis. (**A**) Distribution of gene expression relative values in paired non-varicose vs. varicose vein segments in patients of the whole sample; (**B**) distribution of gene expression relative values in non-varicose and varicose vein segments according to sex; (**C**) scatter plot and the corresponding regression line for the relationship between the dependent (gene expression) variable and independent (sex) variable; (**D**) multiple linear regression analysis results of the *TIMP1* gene expression. The box borders show the interquartile range, the horizontal line inside it indicates the median, and the whiskers show the maximum and minimum values; NV—non-varicose vein; VV—varicose vein; MLR—multiple linear regression; * *p*-value < 0.05, ** *p*-value < 0.01 (Wilcoxon test (**A**), Student’s *t*-test (**B**)); ^†^ all parameters (independent predictor variables) applied together: sex, age, BMI, VVD manifestation, CEAP class, height, VVD duration (data are provided only for sex); ^#^ sex is the only independent predictor variable; Beta—regression coefficient; R—coefficient of multiple correlation (the positive square root of the coefficient of multiple determination); *p* (Beta)—*p*-value of Beta; *p* (R)—*p*-value of R; Beta and R > |±0.25| are displayed in blue at *p*-value > 0.05; Beta and R > |±0.25| are displayed in red at *p*-value < 0.05; 0.05 < *p*-values < 0.1 are displayed in blue; *p*-values < 0.05 are displayed in red; r—correlation coefficient; r > |±0.5| are displayed in red; the number of patients of different sexes is indicated in the legend in the brackets; empty graphs (“–”) mean that MLR analysis results are not available (there were no variables in the regression equation).

**Figure 5:**
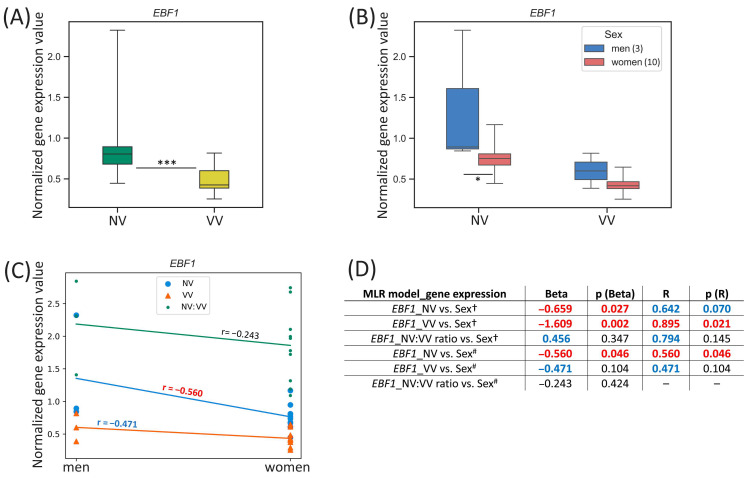
*EBF1* gene expression (mRNA level) data analysis. (**A**) Distribution of gene expression relative values in paired non-varicose vs. varicose vein segments in patients of the whole sample; (**B**) distribution of gene expression relative values in non-varicose and varicose vein segments according to sex; (**C**) scatter plot and the corresponding regression line for the relationship between the dependent (gene expression) variable and independent (sex) variable; (**D**) multiple linear regression analysis results of the *EBF1* gene expression. The box borders show the interquartile range, the horizontal line inside it indicates the median, and the whiskers show the maximum and minimum values; NV—non-varicose vein; VV—varicose vein; MLR—multiple linear regression; *** *p*-value < 0.001, * *p*-value < 0.05 (Wilcoxon test (**A**), Student’s *t*-test (**B**)); ^†^ all parameters (independent predictor variables) applied together: sex, age, BMI, VVD manifestation, CEAP class, height, VVD duration (data are provided only for sex); ^#^ sex is the only independent predictor variable; Beta—regression coefficient; R—coefficient of multiple correlation (the positive square root of the coefficient of multiple determination); *p* (Beta)—*p*-value of Beta; *p* (R)—*p*-value of R; Beta and R > |±0.25| are displayed in blue at *p*-value > 0.05; Beta and R > |±0.25| are displayed in red at *p*-value < 0.05; 0.05 < *p*-values < 0.1 are displayed in blue; *p*-values < 0.05 are displayed in red; r—correlation coefficient; r > |±0.3| is displayed in blue; r > |±0.5| is displayed in red; the number of patients of different sexes is indicated in the legend in the brackets; empty graphs (“–”) mean that MLR analysis results are not available (there were no variables in the regression equation).

To further identify and characterize relationships among sex and multiple factors (relative mRNA level of a gene in NV or VV, or their ratio), multiple linear regression (MLR) analysis was performed (for the details, see the Section 2. Two kinds of MLR were implemented to the gene expression data: (1) all additional parameters available (such as age, BMI, VVD manifestation, CEAP class, height, VVD duration) were applied simultaneously with the main studied independent predictor variable—sex (to take into account their possible joint influence) vs. dependent variables (gene expression relative values in series: for NV, VV, or their ratio); (2) only the main studied independent predictor variable—sex—was applied vs. dependent variables; gene expression relative values for NV, VV, and their ratio. The data tables on both kinds of MLR analyses for all the models used is presented in Figure 3D, Figure 4D and Figure 5D in the text below for the *STK38L*, *TIMP1*, and *EBF1* genes or in Supplementary Table S2 for other genes investigated. According to the multicollinearity diagnostics performed to ensure regression model stability, none of our regression models had the multicollinearity issue since VIFs and GVIFs were below their thresholds (the results are shown in the last column of Supplementary Table S2).

Additionally, using MATCH Suite (release 3.1 by geneXplain) software package, we performed gene regulation analysis for the aforementioned genes demonstrating expression differences in veins in a sex-specific manner, as well as for some other genes with a similar tendency. The goal of the analysis was to reveal sex hormone-related transcription factor binding sites (TFBS) for the studied genes and to identify the transcription factors regulating those genes through their regulatory regions (promoter and associated enhancers and silencers). Specifically, we were interested in TFBS for transcription factors themselves acting as nuclear receptors for their ligands—sex hormones. These receptors include androgen receptors (AR), estrogen receptors (ER), and progesterone receptors (PR). Such TFBS are crucial for regulating gene expression and are located in the regulatory regions of genes that are responsive to sex hormones like androgen, estrogen, and others. The results of the analysis are shown in Table 2 and Supplementary Table S3.

#### 3.3.1. *STK38L*

For the *STK38L* gene located on the chromosome 12, we observed the differences in its expression between men and women in both sample subsets—NVs and VVs: it was higher in NVs and VVs of women 3.11-fold (*p*-value = 0.014) and 2.69-fold (*p*-value = 0.024), correspondingly (Figure 3B).

One can see that *STK38L* gene expression in VV moderately correlated with sex (r = 0.449, *p*-value = 0.024) (Figure 3C). According to MLR analysis performed, sex had a direct effect on the *STK38L* relative mRNA level in VV when it was applied as an independent variable simultaneously with other parameters (Beta = 0.504, *p* (Beta) = 0.013; R = 0.516, *p* (R) = 0.033) (Figure 3D). Moreover, the addition of other independent variables in MLR did not change its single effect for VV (Beta = 0.449, *p* (Beta) = 0.024; R = 0.449, *p* (R) = 0.024). Sex tended to have a weak effect on the *STK38L* relative mRNA level in NV but it did not reach statistical significance of Beta and R coefficients.

In a search for sex hormone-related TFBS within regulatory regions of the *STK38L* gene (see Table 2), we revealed 78 binding sites (two of them in the promoter, and others in the enhancers, with affinity score = 12.20, affinity *p*-value = 4.88 × 10^−5^) for androgen receptor (AR) and progesterone receptor (PR), as well as 43 binding sites (one of them in the promoter, and others in the enhancers, with affinity score = 10.35, affinity *p*-value = 5.68 × 10^−4^) for estrogen receptor 1 (ER-alpha), estrogen receptor 2 (ER-beta), estrogen-related receptor alpha (ERR1), estrogen-related receptor beta (ERR2), and estrogen-related receptor gamma (ERR3) pointing to sex hormones’ effect on this gene’s expression in veins.

#### 3.3.2. *TIMP1*

For the *TIMP1* gene located on the chromosome X, we observed the difference in its expression between men and women in VV sample subset: it was 1.32-fold higher (*p*-value = 0.031) in VVs of men (Figure 4B). Additionally, when comparing the *TIMP1* expression in VV/NV paired samples’ ratio between men and women, we found that it tended to be 2.49-fold higher (*p*-value = 0.077) in men compared to women.

One can see that *TIMP1* gene expression in VV strongly correlated with sex (r = −0.612, *p*-value = 0.026) and for VV/NV ratio it had a tendency to moderate correlation (r = −0.5127, *p*-value = 0.073) (Figure 4C). According to linear regression analysis performed, when sex was the only independent predictor variable applied, sex had a strong reverse effect on the *TIMP1* relative mRNA level in VV (Beta = −0.612, *p* (Beta) = 0.026; R = 0.612, *p* (R) = 0.026) (Figure 4D). Sex tended to have a moderate reverse effect on the VV/NV ratio of the *TIMP1* expression but it did not reach statistical significance of Beta and R coefficients. The addition of other independent predictor variables in MLR changed its single effect for VV/Beta coefficient was tiny and did not reach statistical significance despite high R = 0.867 at *p* (R) = 0.004, meaning that additional parameters other than sex made more substantial relative contribution in the prediction of the dependent variable (*TIMP1* expression in VV).

In a search for sex hormone-related TFBS within regulatory regions of the *TIMP1* gene (see Table 2), we revealed only five binding sites within its enhancer (with affinity score = 8.44, affinity *p*-value = 1.85 × 10^−2^) for AR and PR, indicating that besides sex hormones’ effect, another way of sex influence on this gene’s expression in veins may take place since it is located directly on the X chromosome.

#### 3.3.3. *EBF1*

For the *EBF1* gene located on the chromosome 5, we observed the difference in its expression between men and women in NV sample subset: it was 1.77-fold higher (*p*-value = 0.046) in NVs of men (Figure 5B). However, a tendency for its sex-specific expression in VV sample subset was also detected: it was 1.39-fold higher (*p*-value = 0.104) in VVs of men compared to women.

One can see that *EBF1* gene expression in NV moderately correlated with sex (r = −0.560, *p*-value = 0.046) (Figure 5C). According to the MLR analysis performed, sex had a strong reverse effect on the *EBF1* relative mRNA level in NV (Beta = −0.659, *p* (Beta) = 0.027) pointing to a considerable relative contribution of sex compared to other independent parameters applied (since R = 0.642, *p* (R) = 0.070) in the prediction of the dependent variable (*EBF1* expression in NV) (Figure 5D). An exceedingly reverse effect of sex on the *EBF1* relative mRNA level in VV was observed when it was applied as an independent variable simultaneously with other parameters (Beta = −1.609, *p* (Beta) = 0.002; R = 0.895, *p* (R) = 0.021). Even though Beta coefficient appeared to be greater than 1 it was not excluded from the analysis because that could not be due to multicollinearity of sex variable with other parameters studied. Sex as an individual predictor was examined further and indicated that it was a moderate predictor for the *EBF1* expression in NV (Beta = −0.560, *p* (Beta) = 0.046; R = 0.560, *p* (R) = 0.046).

In a search for sex hormone-related TFBS within regulatory regions of the *EBF1* gene (see Table 2), we revealed 59 binding sites (two of them in the promoter, and others in the enhancers, with affinity score = 4.39, affinity *p*-value = 6.88 × 10^−5^) for PR and AR, as well as 52 binding sites in the enhancers of this gene (with affinity score = 6.06, affinity *p*-value = 3.6 × 10^−3^) for ER-alpha, ER-beta, ERR1, ERR2, and ERR3, which points to sex hormones’ effect on this gene’s expression in veins.

Additionally, we also performed a search for sex hormone-related TFBS within the regulatory regions of the genes demonstrating tendencies for sex-specific expression (see Supplementary Table S3): downregulated *VCL* (1.31-fold lower in VVs of women, *p*-value = 0.082) and *PPP1R12B* (1.95-fold lower in NVs of women, *p*-value = 0.081), as well as upregulated *ITGA5* (1.32-fold lower in VVs of women, *p*-value = 0.061; 1.44-fold lower in VV/NV ratio of women, *p*-value = 0.090). As a result, 29 (5 of them in the promoter, and others in the enhancers), 14 (2 of them in the promoter, and others in the enhancers), and 16 (1 of them in the promoter, and others in the enhancers) TFBS for AR and PR were found for the *VCL*, *PPP1R12B*, and *ITGA5* genes, respectively, as well as 23 (in the enhancers), 6 (in the enhancers), and 28 (2 of them in the promoter, and others in the enhancers) TFBS for ER-alpha, ER-beta, ERR1, ERR2, and ERR3, were found for the *VCL*, *PPP1R12B*, and *ITGA5* genes, respectively, on average, with lower affinity values compared to the *STK38L* and *EBF1* genes demonstrating sex-specific expression in veins.

## 4. Discussion

The necessity for differential treatment of various diseases depending on sex has long been reported. Sex has been suggested for considering as an important variable to study the pathogenesis of certain diseases [20]. In chronic venous diseases, sex differences are also of high significance to clinical research and practice. As such, sex and age are determining factors for chronic venous disease and influence the risk by 10.7% [43,44]. Studies of changes in gene expression provide insights into the molecular mechanisms implicated in the transition from a healthy to a disease state and can be used to identify patients at higher risk for particular medical conditions, higher symptom burden, and adverse consequences associated with various treatments [45]. It was demonstrated that changes in the expression level of genes (in most cases, with nonlinear relationship between expression and a phenotype) may predictably alter genetic interactions [46]. During critical developmental stages, human body systems demonstrate sex-specific gene expression that may be regulated by hormone-responsive elements related to those genes and could influence the incidence and manifestation of certain diseases across sexes [20]. In the case of VVs, venous transformation can be considered as the formation of a modified organ, when critical stages of this process governed by sex-specific gene expression also take place.

In the present study, first of all, we validated our previous microarray data [22] for a set of genes differentially expressed in VVs (Table 1), thereby confirming their plausible participation in the remodeling of the venous wall. Then, we evaluated sex-specific differences in the expression of these genes within NV and VV sample (or their paired ratio) subsets separately. Certain sex-related differences have been observed for the expression of the *STK38L* (higher in NVs and VVs of women), *TIMP1* (higher in VVs of men, tended to be higher in VV/NV ratio of men) and *EBF1* (higher in NVs of men, tended to be higher VVs of men) genes (Figure 3B, Figure 4B and Figure 5B), compared to other genes investigated (Supplementary Figures S1B–S20B). However, the tendencies for sex-specific expression were detected for the *VCL* (higher in VVs of men), *PPP1R12B* (higher in NVs of men), and *ITGA5* (higher in VVs and VV/NV ratio of men).

The results of MLR analyses enabled us to extract additional information regarding the association of sex (along with various parameters—other predictor variables) with the expression level of the studied genes in veins. As such, sex (either as the only independent variable or in combination with other parameters) directly affected the *STK38L* expression in VVs (being higher in women) suggesting its sex-dependent involvement in the development of the disease. For the *TIMP1* gene, sex (only as a separate independent variable) obviously affected its expression in VVs (being higher in men). Sex also influenced the *EBF1* mRNA level in NVs. Since the *EBF1* was downregulated in VVs and its expression was 1.77-fold lower (before varicose transformation) in NVs of women compared to men, it is logical to assume the existence of systemic effects of this gene’s product on the development of VVs and additional evidence of the female sex as a risk factor for this pathology.

Sex differences in gene expression vary not only across the genome but between individuals, which was demonstrated in our data on gene expression with interindividual variability. The study by Oliva et al. highlighted that overlapping distributions of gene expression between the sexes could be the result of not strictly dimorphic interindividual variation [47,48]. It was shown that *STK38L* (serine/threonine kinase 38 like) gene product promotes cardiac fibroblast activation and proliferation, and its increased expression is closely associated with atrial fibrillation characterized by atrial fibrosis and TGF-β1-induced myocardial fibrosis [49]. Additionally, *STK38L* is involved in apoptosis signaling, alignment of mitotic chromosomes, controlling centrosome duplication, and G1/S transition, promoting G1 progression [50]. It is noteworthy that *TIMP1* gene is located on the X chromosome and therefore can escape random X inactivation, which may result in higher expression levels of this gene in women than in men [51]; moreover, this gene inactivation is polymorphic in human females, which results in predisposition of some women to this gene’s expression [52], and its transcription is highly inducible in response to many cytokines and hormones. Notwithstanding this, according to our data for veins, we observed a higher expression level of this gene in VVs of men compared to women, as well as a tendency for higher VV/NV expression ratio in men. The explanation for this phenomenon could be that men have an always-active single X chromosome, while women have two X chromosomes but a randomly inactive one, causing mosaic expression and often reduced gene dosage per cell for many X-linked genes, also taking into account tissue-specific distribution of the female/male gene fold ratio for some X-linked genes (ranging from 3 to 18% of genes with higher expression in males depending on tissue) [53]. Interestingly, sex differences in extracellular matrix production and remodeling related, in particular, to matrix metalloproteinases and their tissue inhibitors were demonstrated [54]. The important regulatory effect of the *TIMP1*, being expressed in the liver endothelial and stellate cell populations, on the formation and degradation of liver fibrosis was observed [55], as well as its participation in ventricular remodeling caused by hypertension, leading to myocardial fibrosis [56]. The *EBF1* gene was prioritized as one of the most likely causative genes for VVs development [57]. Its product is a transcription factor that promotes B cell and pericyte cell differentiation (being a bona fide pericyte marker) [58], is involved in blood pressure and hypertension, and is associated with carotid intimal thickness [59], cardiovascular, and metabolic risk [60,61], as well as immune response/inflammation [57]. The mechanotransduction protein vinculin, encoded by the *VCL* gene (its defects cause dilated cardiomyopathy), is important for the endothelial barrier due to the recruitment of vinculin to AJs protein, which results in strengthening the endothelial cell–cell junctions in blood vessels and preventing developing vessels from vascular leakage [62]. As for the *PPP1R12B* gene coding for myosin phosphatase 1 regulatory subunit 12B (that undergoes insulin-stimulated phosphorylation [63]), its intronic variant (rs1819043) is directly involved in the reproductive system and associated with sex ratio [64], and another variant (rs697455) serves as a sex-different autosomal marker [65]. The *PPP1R12B* was identified as a key biomarker for abdominal aortic aneurysm prediction: its downregulation in this disease was linked to increased N6-methyladenosine levels [66]. It is noteworthy that environmentally altered *PPP1R12B* expression (in animal models) sex-dependently involved motivation-related brain regions [67]. The *ITGA5* gene was shown to be among the four common genes of all three important processes (‘extracellular matrix organization’, ‘cell adhesion’, ‘blood vessel morphogenesis’) largely activated in VV condition, and its product—integrin alpha-5 protein—was identified as a potential primary master regulator of VV pathogenesis [22].

In terms of genetic mechanisms, there are two general models (not necessarily exclusive of one another) known to explain sex differences in gene expression: (1) when hormones in men and women differentially influence various genes’ expression level and (2) when rate-limiting step in different pathways is encoded/influenced by an X- or Y-chromosome-linked gene with dosage differences between men and women [68]. Besides possible mechanisms exerting sex differences in VVs, such as skewed X chromosome inactivation and genes escaping it, cellular mosaicism, and miRNAs encoded on the X chromosome, the most studied have been effects of sex hormones and their receptors. These receptors, specifically distributed on the cell surface according to sex, may define differences in susceptibility to disease, the average age of onset, and response to therapy [69]. Due to both genomic and nongenomic effects of sex hormones on the endothelium and smooth muscle cells, the vascular effects of sex hormones could be different in the two sexes [69]. Regarding the contribution of hormonal regulation in the process of varicose transformation, a possible causal relationship between sex steroids (elevated serum estradiol and testosterone levels along with downregulation of hormonal receptors and enzymes involved in steroid metabolism) and VVs was shown in men [70]. In addition, greater venous distensibility and VVs in menopausal women have been associated with high estrogen levels [21]. How hormones affect molecular changes occurring within the venous wall, remains to be established considering many factors implicated, including differences in the stimulation or inhibition of cell populations by sex hormones, differences in the regulation of vascular tone and synthesis of the extracellular matrix products, as well as hormones’ and their receptors’ actions in numerous pathways [69]. For instance, Fan et al. have partly explained the sex-stratified prevalence of VVs by the greater odds ratio of higher serum sex hormone-binding globulin (SHBG) levels in women, compared to men [71]. While discussing sex differences in vascular endothelial function regarding COVID-19, Kitselman et al. called attention to the existence of a testosterone-inducible element located in the promoter of the host transmembrane serine protease gene—*TMPRSS2*, which explains sex-based differences in the immune response [51]. They also mentioned the potential reason for the observed sex differences in myalgic encephalomyelitis chronic fatigue syndrome (ME-CFS)—a recurrent chronic disease predominantly affecting females (as in case of varicose veins) and linked to endothelial dysfunction and the immune system: the modulation of cytokine gene expression by steroid hormones, such as estrogen. In the context of the vein-specific inflammation component in the process of varicose transformation, it is worthy of noting about direct estrogen-related stimulation of IL-1 production by macrophages [51].

Besides the hormonal hypothesis, epigenetics as the unifying mechanism in explaining sex-specific differences in disease susceptibility has been amply demonstrated [72]. In this regard, a large-scale meta-analysis with integration of the DNA methylome and transcriptome in human skeletal muscle identified sex-specific genes associated with muscle contraction, substrate metabolism and anatomical structure-related pathways, suggesting DNA methylation and gene expression differences between men and women in skeletal muscle are functionally linked, and that hormone-related transcription factor binding sites (TFBS) and muscle fiber type proportions underlie those differences [73]. This has also been in tune with our sample set: we observed sex-specific differences in anatomical manifestation of CVeD (Figure A1) and additionally proved that compared to men, a larger percentage of women suffered from hypothyroidism and autoimmune diseases (Figure A2)—the conditions to which they had a higher susceptibility [20] since their vascular dysfunction is often characterized by an imbalance of thyroid hormones. Sex-related features of insulin resistance and vascular calcification were also observed in women [20].

Additional analysis on the regulatory regions of the studied genes helped us uncover meaningful biological insights on sex differences in the genetic regulation of gene expression and gain a more thorough view of the sex-related molecular basis of VVs. Indeed, the integration of regulatory region motif searches for sex hormone-related TFBS with signaling pathway knowledge available in the geneXplain platform allowed us to move from a descriptive list of gene expression changes to a testable model of why those changes occurred. As a result, we identified matrices (site models) regulating the input genes through sex hormone-inducible transcription factors (AR, PR, ER-alpha, ER-beta, ERR1, ERR2, ERR3) (see Table 2 and Supplementary Table S3). As for the *STK38L*, 78 (2 in the promoter; 76 in the enhancers) TFBS for AR and PR, and 43 (1; 42) TFBS for ER-alpha, ER-beta, ERR1, ERR2, and ERR3 were revealed. As for the *TIMP1*, 5 (0; 5) TFBS for AR and PR were found. As for the *EBF1*, 59 (2; 57) TFBS for AR and PR, and 52 (0; 52) TFBS for ER-alpha, ER-beta, ERR1, ERR2, ERR3 were identified. This indicates that *STK38L* and *EBF1* expression in veins is affected by sex hormones while the expression of the *TIMP1*, located on the X chromosome, is most likely affected by sex through different mechanisms. Among many differentially expressed genes found on the X chromosome, only 4% were with sex-differential expression [48]. Another prominent example: in a search for sex hormone-related TFBS within regulatory regions of the *Mn-SOD* gene, we did not reveal any, which may indicate that sex hormones do not affect this gene’s expression in veins.

Our data are consistent with the fact that the vast majority of AR binding sites is located in enhancers, with few binding sites found in promoters; moreover, clinical AR binding sites display remarkable plasticity and reprogramming during disease progression so that AR may gain many more additional binding sites [74]. Several studies have shown that the epigenetic landscape of enhancers is intricately organized by several regulatory protein complexes during development into a predetermined pattern [75]. Using STARR-seq, three different classes of binding sites constituting inducible, inactive, and constitutive enhancers, were revealed. While 81% of AR binding sites did not demonstrate any enhancer activity and approximately 12% exhibited constitutive enhancer activity independently of AR binding, only 7% of the regions showed AR-activated or inducible enhancer activity [74]. Also, a comprehensive genome-wide analysis revealed that enhancer activity is strongly influenced by the genomic location of a full estrogen response element and that active ER binding site clusters (being associated with more pronounced expression changes) are more likely to be found nearby regulated genes [76]. For instance, though there are many ER binding site clusters throughout the genome, it was shown that most genes do not have an active cluster within 100 kb that resembles particular enhancers [76].

The latter is true for our data (see Supplementary Table S3). Compared to the genes which demonstrate sex-specific expression in veins, it may seem that some other genes’ regulatory regions contain even more sex hormone-inducible TFBS, such as in case of the *ABCA1* gene: it has 156 (9; 147) TFBS for AR and PR, as well as 53 (0; 53) TFBS for ER-alpha, ER-beta, ERR1, ERR2, and ERR3. Nevertheless, merely a large number of particular TFBS does not guarantee sex-specific regulation of a gene because only 37 out of 147 and 15 out of 53 TFBS in enhancers, correspondingly, are located within 100 kb of the *ABCA1* gene’s promoter, meaning that the majority of them are not sex hormone-inducible. Moreover, the *ABCA1* was absent among sex-biased genes in the data presented within the scrutinizing work by Oliva et al. who provided an extensive characterization of sex differences in the human (44 tissue sources) transcriptome and its genetic regulation [47].

Oliva et al. concluded that sex-specifically expressed genes are involved in diverse biological functions and not primarily driven by tissue-specific gene expression; so that sex-related distribution of epigenetic marks (such as H3K27me3), the enrichment of TFBS in sex-biased gene promoters, along with the high tissue specificity of sex-biased gene expression, implies the latter is mediated by specific transcription factors [47]. According to their data, we can conclude that sex-specific regulation of a gene strongly depends on the tissue/organ where it is expressed: for instance, it is very true for the *TIMP1* gene which expression is female-biased particularly in artery_tibial (enrichment *p*-value = 2.05 × 10^−13^), brain_cortex (enrichment *p*-value = 2.39 × 10^−8^), brain_hippocampus (enrichment *p*-value = 3.21 × 10^−9^). Therefore, we can somehow unambiguously extrapolate that to vein_saphenous (also attributing to the ‘blood vessel’ tissue group) investigated in our study. As for the limitations of the work by Oliva et al., over two-thirds of the samples were from men, meaning that studies were unevenly statistically powered to detect sex differences [48].

Limitations of the present study include the small study sample with a fewer number of men than women, which may not be representative of all men who develop VVs. The interpretability of these results may be limited by the complex interrelations of the expression of genes investigated with other associated conditions and comorbidities. Certainly, the sex-specific regulation of those genes in varicose and normal superficial veins requires further clinical and laboratory studies.

Last but not least, there may be sex differences in pharmacological effects of any drugs due to disparities in pharmacokinetics and pharmacodynamics of their absorption, distribution, metabolism, and excretion. Therefore, this knowledge when being applied could be essential to ensure the proper effects of drugs [77]. As an example, sex differences in aspirin treatment were experimentally proved: it was less effective in preventing retinal ischemia in female than in male diabetic rats [78]. Additionally, there could be modulating effects of some vasoactive substances on hormonal responsiveness: in case of resveratrol, such differences were observed between males and females [79]. We should also consider sex differences regarding VVs in order to move forward towards the personalized genomic medicine application.

## 5. Conclusions

The present study is one of the few attempts, to the best of our knowledge, to assess how male or female sex may contribute to the changes to gene expression profiles in the vein wall during varicose transformation. After verification of altered expression of a set of genes in VVs, we have shown sex-specific expression in veins for the *STK38L*, *TIMP1*, and *EBF1* genes, as well as some sex-related tendencies for other genes differentially expressed in VVs, and that sex was a predictor for their expression. Furthermore, using a search for binding sites for transcription factors—sex hormone receptors—within the regulatory regions of the studied genes, we obtained insights on their sex-specific regulation. To further delve into the mechanisms of these genes’ sex-specific regulation it is necessary to reveal complex transcription factor interactions in promoters and enhancers, which may help us understand how gene expression patterns define cell identity and disease. Understanding sex features for the development of VVs may provide the foundation for preventative measures, beneficial treatments, and curative therapies.

## Figures and Tables

**Table 1:** Differentially expressed genes confirmed by RT-qPCR.

Gene	Gene Description	Gene Expression Fold Change ^†^ (95% CI) ^§^	*p*-Value *
*ABCA1*	ATP binding cassette subfamily A member 1	(↓) 3.12 (2.38–4.09)	0.00024
*AXL*	AXL receptor tyrosine kinase	(↓) 1.48 (1.18–1.88)	0.00415
*CALU*	calumenin	(↑) 1.89 (1.40–2.56)	0.00004
*CASZ1*	castor zinc finger 1	(↑) 1.28 (1.00–1.63)	0.03588
*CCL2*	C-C motif chemokine ligand 2	(↑) 1.94 (1.37–2.75)	0.00868
*CHRDL2*	chordin like 2	(↑) 2.15 (1.37–3.39)	0.00464
*COL15A1*	collagen type XV alpha 1 chain	(↑) [1.46] (1.21–1.76)	0.00171
*COMP*	cartilage oligomeric matrix protein	(↑) 2.19 (1.58–3.05)	0.00014
*EBF1*	EBF transcription factor 1	(↓) 1.81 (1.49–2.20)	0.00024
*EFEMP1*	EGF containing fibulin extracellular matrix protein 1	(↑) 2.01 (1.29–3.14)	0.00464
*GPI*	glucose-6-phosphate isomerase	(↑) 1.57 (1.29–1.92)	0.00015
*ITGA5*	integrin subunit alpha 5	(↑) 1.42 (1.20–2.46)	0.02186
*MFAP5*	microfibril associated protein 5	(↑) 1.60 (1.12–2.28)	0.00664
*Mn-SOD*	superoxide dismutase 2	(↓) 1.53 (1.33–1.83)	0.00101
*MYO18B*	myosin XVIIIB	(↑) 1.67 (1.17–2.39)	0.00745
*MYOD1*	myogenic differentiation 1	(↓) 1.53 (1.27–1.81)	0.00298
*PLA2G2A*	phospholipase A2 group IIA	(↑) 3.80 (2.36–6.10)	0.00004
*PPP1R12B*	protein phosphatase 1 regulatory subunit 12B	(↓) 1.56 (1.11–2.19)	0.02148
*STK38L*	serine/threonine kinase 38 like	(↑) 2.04 (1.36–3.04)	0.00203
*SULF1*	sulfatase 1	(↑) 1.46 (1.22–1.76)	0.00018
*TIMP1*	TIMP metallopeptidase inhibitor 1	(↑) [1.58] (1.15–2.17)	0.00464
*TNC*	tenascin C	(↑) 1.36 (1.13–1.74)	0.03978
*VCL*	vinculin	(↓) 1.30 (1.21–1.49)	0.00006

^†^ The mRNA fold changes between paired ‘varicose’ and ‘non-varicose’ vein segments of a patient: VV/NV ratio for upregulated (↑) genes or NV/VV ratio for downregulated (↓) genes; ^§^ 95% CI: range of values (lower–upper) of the 95% confidence interval of the ratio; * *p*-value < 0.05, according to Wilcoxon signed-rank test performed using qBase+ (version 3.2) or STATISTICA (version 8.0) software.

**Table 2:** Sex hormone-related TFBS within regulatory regions of the genes demonstrating differences (*p*-value < 0.05) and tendencies (0.05 < *p*-value < 0.1) for sex-specific expression in veins.

Gene	Chromosome	Number of TFBS ^†^: Total (in the Promoter; in the Enhancer(s))	Affinity Score ^‡^	Affinity *p*-Value ^§^
*STK38L*	12	78 (2; 76) for AR and PR	12.20	4.88 × 10^−5^
		43 (1; 42) for ER-alpha, ER-beta, ERR1, ERR2, ERR3	10.35	5.68 × 10^−4^
*TIMP1*	X	5 (0; 5) for AR and PR	8.44	1.85 × 10^−2^
		–	–	–
*EBF1*	5	59 (2; 57) for AR and PR	4.39	6.88 × 10^−5^
		52 (0; 52) for ER-alpha, ER-beta, ERR1, ERR2, ERR3	6.06	3.6 × 10^−3^
*VCL*	10	29 (5; 24) for AR and PR	9.33	1.35 × 10^−3^
		23 (0; 23) for ER-alpha, ER-beta, ERR1, ERR2, ERR3	9.77	1.07 × 10^−3^
*PPP1R12B*	1	14 (2; 12) for AR and PR	6.82	1.28 × 10^−2^
		6 (0; 6) for ER-alpha, ER-beta, ERR1, ERR2, ERR3	10.78	1.1 × 10^−3^
*ITGA5*	12	16 (1; 15) for AR and PR	5.1	5.02 × 10^−3^
		28 (2; 26) for ER-alpha, ER-beta, ERR1, ERR2, ERR3	10.39	6.1 × 10^−4^

^†^ TFBS: transcription factor binding sites; transcription factors (sex hormone receptors) associated with the respective matrices: AR—androgen receptor; PR—progesterone receptor; ER-alpha—estrogen receptor 1; ER-beta—estrogen receptor 2; ERR1—estrogen-related receptor alpha; ERR2—estrogen-related receptor beta; ERR3—estrogen-related receptor gamma. ^‡^ Affinity score of the matrix estimates the overall affinity of a binding specificity to each of the studied regulatory regions of the respective gene; it corresponds to its best (minimum) affinity *p*-value among studied regulatory regions. ^§^ Affinity *p*-value: the statistical significance of affinity scores estimated for large sets of random sequences with the dinucleotide composition of human promoters and sequence lengths ranging from 100 to 2500; it is taken from the best matrix (the most relevant and determining the regulation of the analyzed gene) corresponding to the given factors and represents the best affinity *p*-value among all examined regulatory regions of the respective gene.

## Data Availability

Data are contained within the article and Supplementary Materials.

## Supplementary Materials

The following supporting information can be downloaded at https://www.mdpi.com/article/10.3390/biomedicines13102373/s1. Table S1: Primers used in the study; Table S2: Sex-related multiple linear regression analysis results of the expression of genes (listed in the alphabetical order) not included in figures of the main text (due to incomplete significance) and, for the reference (at the end of the Table), genes included in figures of the main text; Table S3: Sex hormone-related TFBS within regulatory regions of the genes (listed in the alphabetical order) not included in Table 2 of the main text; Figures S1–S20: Gene expression (mRNA level) data analysis results for genes (listed in the alphabetical order) not included in figures of the main text (due to incomplete significance).

## Abbreviations

The following abbreviations are used in this manuscript:

AR	Androgen receptor
BMI	Body mass index
cDNA	Complementary DNA
CEAP	Clinical, Etiological, Anatomical, and Pathophysiological (classification)
CVeD	Chronic venous disease
ER-alpha	Estrogen receptor 1
ER-beta	Estrogen receptor 2
ERR1	Estrogen-related receptor alpha
ERR2	Estrogen-related receptor beta
ERR3	Estrogen-related receptor gamma
GO	Gene Ontology
GSV	Great saphenous vein
IQR	Interquartile range
MLR	Multiple linear regression
mRNA	Messenger ribonucleic acid
NVs	Non-varicose veins
PR	Progesterone receptor
RT-qPCR	Reverse transcription quantitative polymerase chain reaction
SSV	Small saphenous vein
TFBS	Transcription factor binding sites
VVD	Varicose vein disease
VVs	Varicose veins
