# Supplementary material for: Sex-Specific Gene Expression Differences in Varicose Veins
Source: Biomedicines. 2025 Sep 27;13(10):2373. doi: 10.3390/biomedicines13102373 (PMC12562233; doi:10.3390/biomedicines13102373)
Supplement: Supplementary file 1 [file biomedicines-13-02373-s001.zip › Supplementary Figures_revised.pdf]

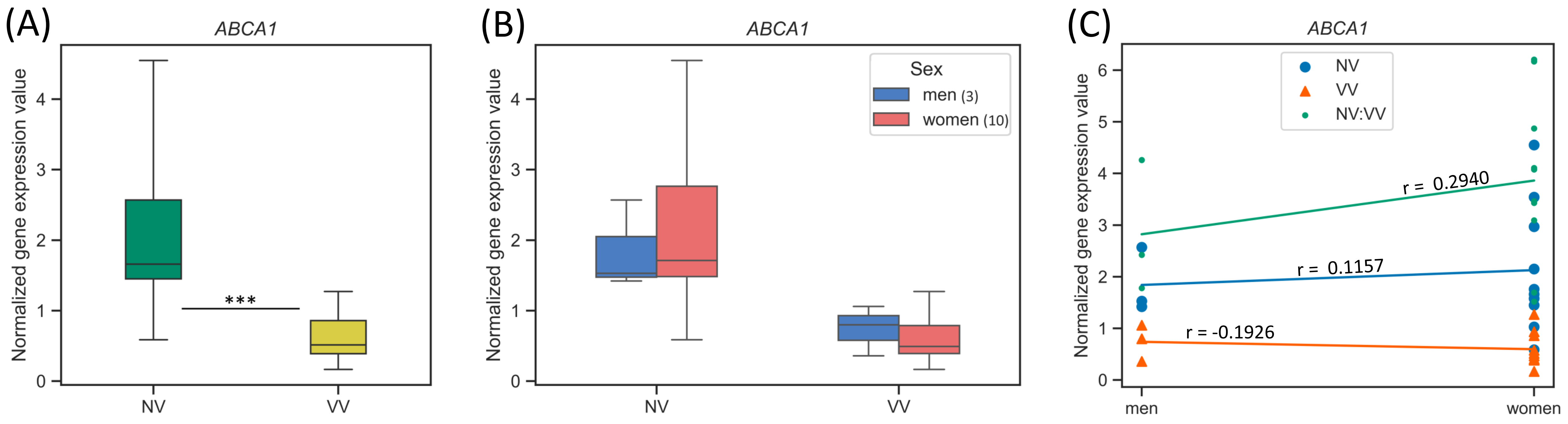

Figure S1. *ABCA1* gene expression (mRNA level) data analysis. (**A**) Distribution of gene expression relative values in paired non-varicose vs. varicose vein segments in patients of the whole sample; (**B**) Distribution of gene expression relative values in non-varicose and varicose vein segments according to sex; (**C**) Scatter plot and the corresponding regression line for the relationship between the dependent (gene expression) variable and independent (sex) variable.

The box borders show the interquartile range, the horizontal line inside it indicates the median, and the whiskers show the maximum and minimum values; NV – non-varicose vein; VV – varicose vein; *** p-value < 0.001 (paired Student's t-test); r – correlation coefficient; the number of patients of different sexes is indicated in the legend in the brackets.

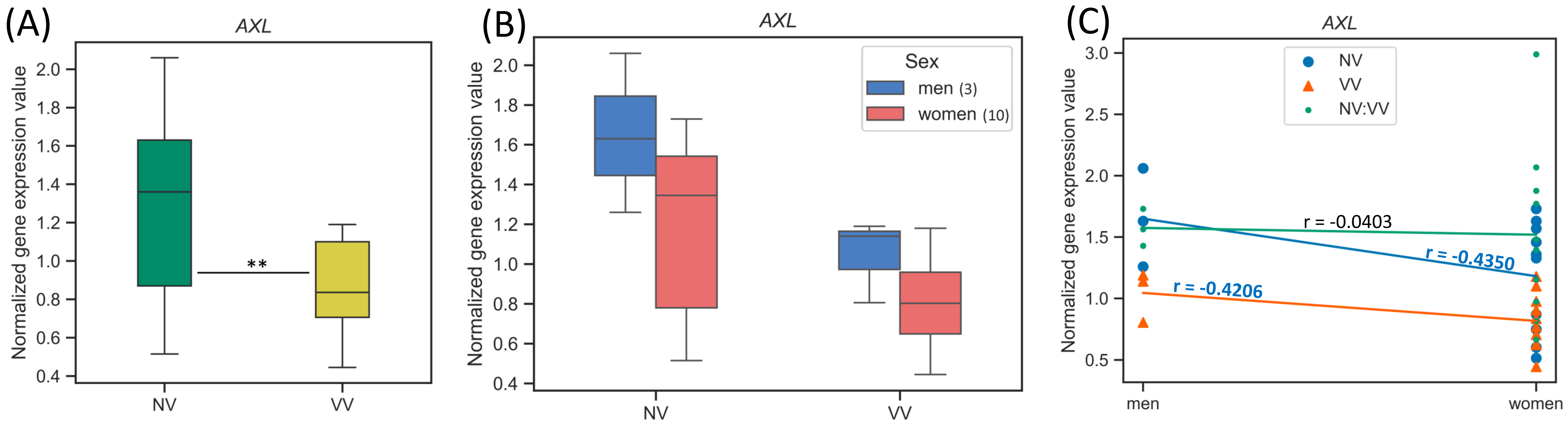

Figure S2. *AXL* gene expression (mRNA level) data analysis. (**A**) Distribution of gene expression relative values in paired non-varicose vs. varicose vein segments in patients of the whole sample; (**B**) Distribution of gene expression relative values in non-varicose and varicose vein segments according to sex; (**C**) Scatter plot and the corresponding regression line for the relationship between the dependent (gene expression) variable and independent (sex) variable.

The box borders show the interquartile range, the horizontal line inside it indicates the median, and the whiskers show the maximum and minimum values; NV – non-varicose vein; VV – varicose vein; ** p-value < 0.01 (Wilcoxon test); r – correlation coefficient; r > |± 0.3| are displayed in blue; the number of patients of different sexes is indicated in the legend in the brackets.

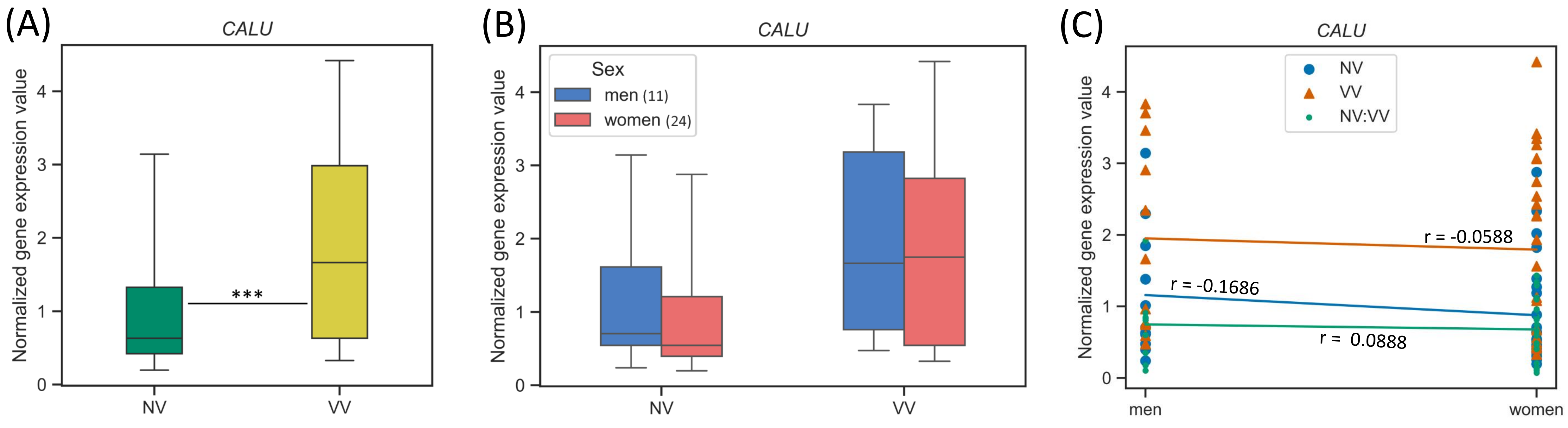

Figure S3. *CALU* gene expression (mRNA level) data analysis. (**A**) Distribution of gene expression relative values in paired non-varicose vs. varicose vein segments in patients of the whole sample; (**B**) Distribution of gene expression relative values in non-varicose and varicose vein segments according to sex; (**C**) Scatter plot and the corresponding regression line for the relationship between the dependent (gene expression) variable and independent (sex) variable.

The box borders show the interquartile range, the horizontal line inside it indicates the median, and the whiskers show the maximum and minimum values; NV – non-varicose vein; VV – varicose vein; *** p-value < 0.001 (Wilcoxon test); r – correlation coefficient; the number of patients of different sexes is indicated in the legend in the brackets.

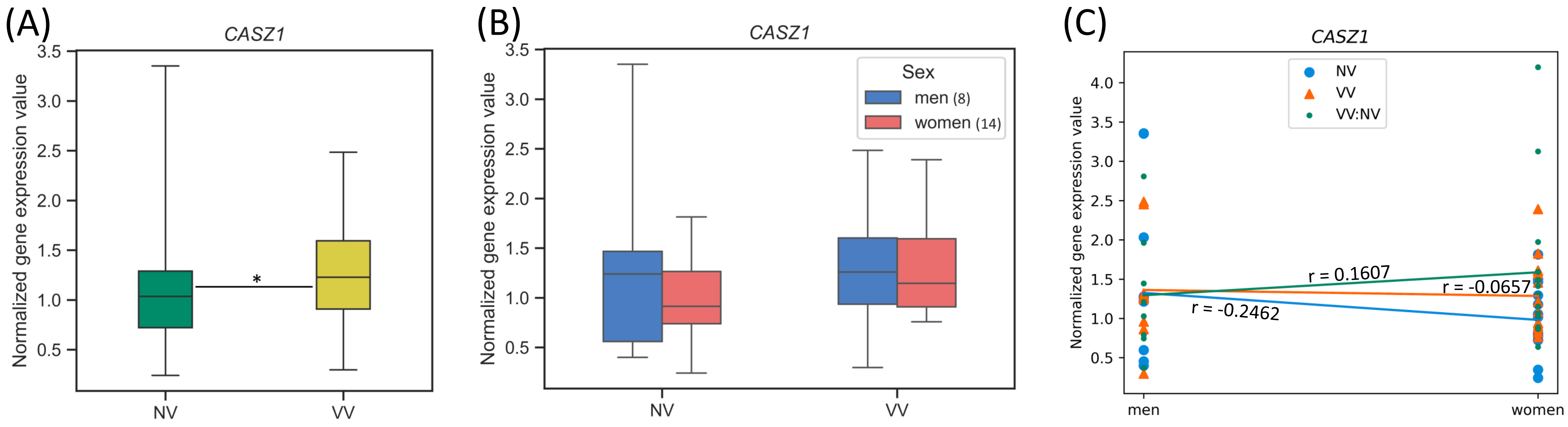

Figure S4. *CASZ1* gene expression (mRNA level) data analysis. (**A**) Distribution of gene expression relative values in paired non-varicose vs. varicose vein segments in patients of the whole sample; (**B**) Distribution of gene expression relative values in non-varicose and varicose vein segments according to sex; (**C**) Scatter plot and the corresponding regression line for the relationship between the dependent (gene expression) variable and independent (sex) variable.

The box borders show the interquartile range, the horizontal line inside it indicates the median, and the whiskers show the maximum and minimum values; NV – non-varicose vein; VV – varicose vein; * p-value < 0.05 (Wilcoxon test); r – correlation coefficient; the number of patients of different sexes is indicated in the legend in the brackets.

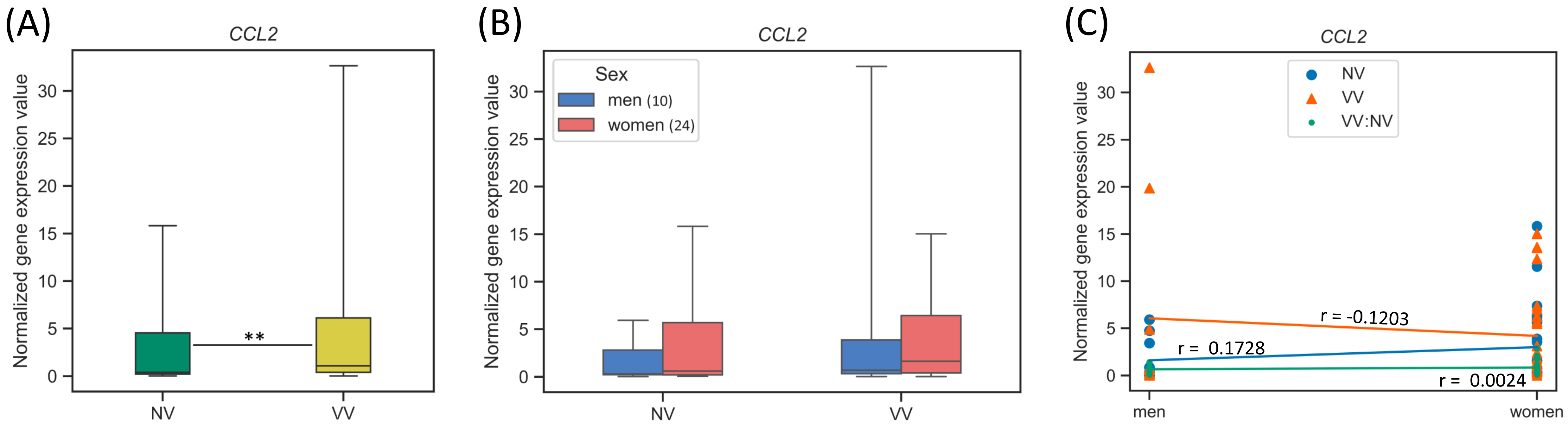

Figure S5. *CCL2* gene expression (mRNA level) data analysis. (**A**) Distribution of gene expression relative values in paired non-varicose vs. varicose vein segments in patients of the whole sample; (**B**) Distribution of gene expression relative values in non-varicose and varicose vein segments according to sex; (**C**) Scatter plot and the corresponding regression line for the relationship between the dependent (gene expression) variable and independent (sex) variable.

The box borders show the interquartile range, the horizontal line inside it indicates the median, and the whiskers show the maximum and minimum values; NV – non-varicose vein; VV – varicose vein; ** p-value < 0.01 (Wilcoxon test); r – correlation coefficient; the number of patients of different sexes is indicated in the legend in the brackets.

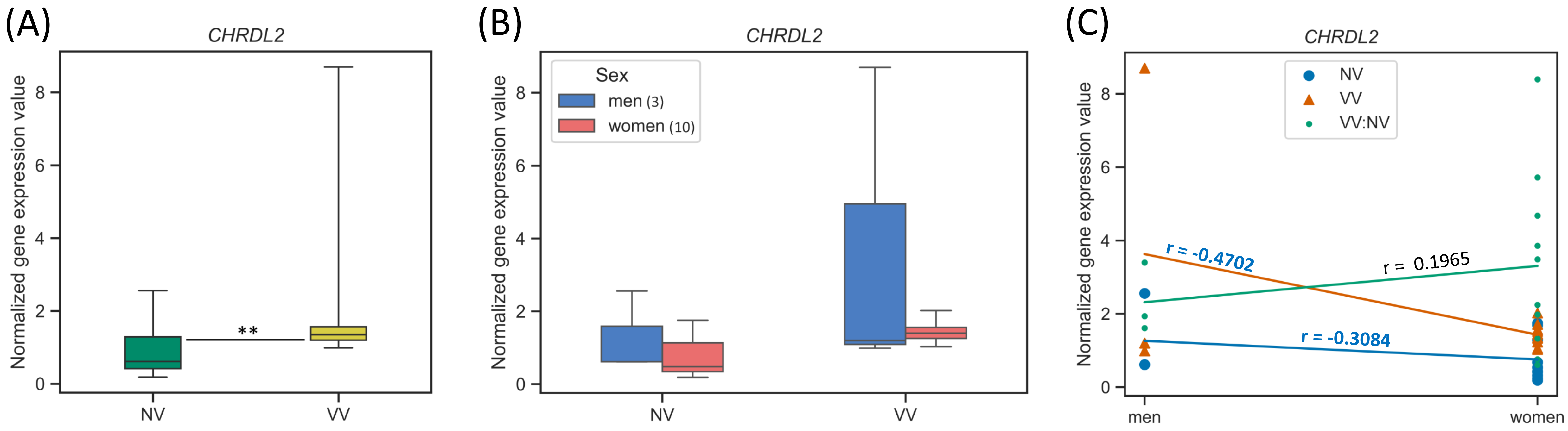

Figure S6. *CHRDL2* gene expression (mRNA level) data analysis. (**A**) Distribution of gene expression relative values in paired non-varicose vs. varicose vein segments in patients of the whole sample; (**B**) Distribution of gene expression relative values in non-varicose and varicose vein segments according to sex; (**C**) Scatter plot and the corresponding regression line for the relationship between the dependent (gene expression) variable and independent (sex) variable.

The box borders show the interquartile range, the horizontal line inside it indicates the median, and the whiskers show the maximum and minimum values; NV – non-varicose vein; VV – varicose vein; ** p-value < 0.01 (Wilcoxon test); r – correlation coefficient; r > |± 0.3| are displayed in blue; the number of patients of different sexes is indicated in the legend in the brackets.

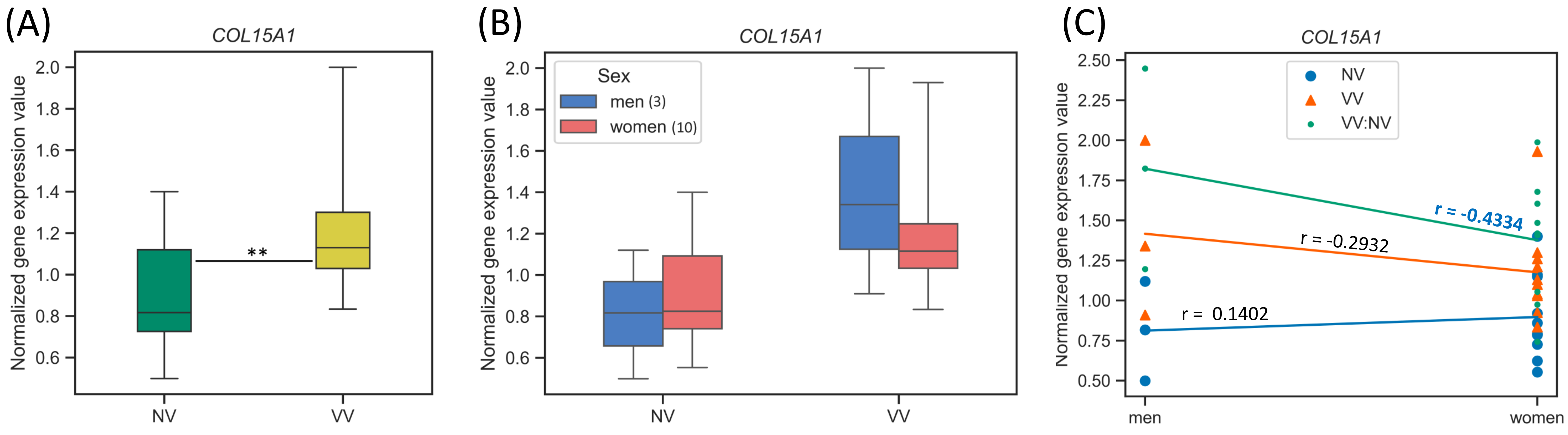

Figure S7. *COL15A1* gene expression (mRNA level) data analysis. (**A**) Distribution of gene expression relative values in paired non-varicose vs. varicose vein segments in patients of the whole sample; (**B**) Distribution of gene expression relative values in non-varicose and varicose vein segments according to sex; (**C**) Scatter plot and the corresponding regression line for the relationship between the dependent (gene expression) variable and independent (sex) variable.

The box borders show the interquartile range, the horizontal line inside it indicates the median, and the whiskers show the maximum and minimum values; NV – non-varicose vein; VV – varicose vein; ** p-value < 0.01 (Wilcoxon test); r – correlation coefficient; r > |± 0.3| is displayed in blue; the number of patients of different sexes is indicated in the legend in the brackets.

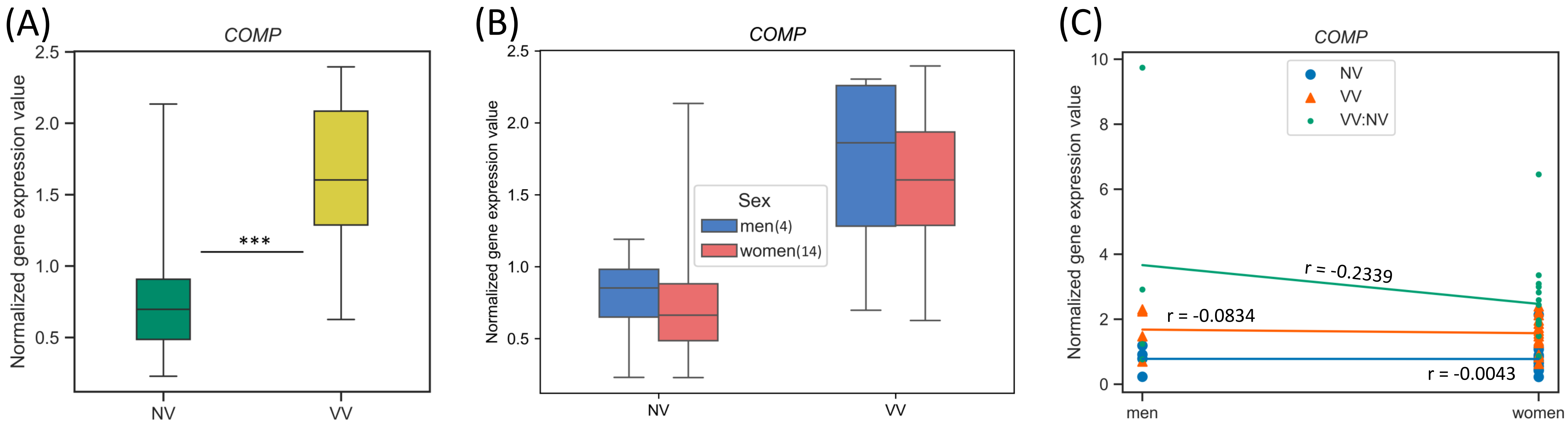

Figure S8. *COMP* gene expression (mRNA level) data analysis. (**A**) Distribution of gene expression relative values in paired non-varicose vs. varicose vein segments in patients of the whole sample; (**B**) Distribution of gene expression relative values in non-varicose and varicose vein segments according to sex; (**C**) Scatter plot and the corresponding regression line for the relationship between the dependent (gene expression) variable and independent (sex) variable.

The box borders show the interquartile range, the horizontal line inside it indicates the median, and the whiskers show the maximum and minimum values; NV – non-varicose vein; VV – varicose vein; *** p-value < 0.001 (Wilcoxon test); r – correlation coefficient; the number of patients of different sexes is indicated in the legend in the brackets.

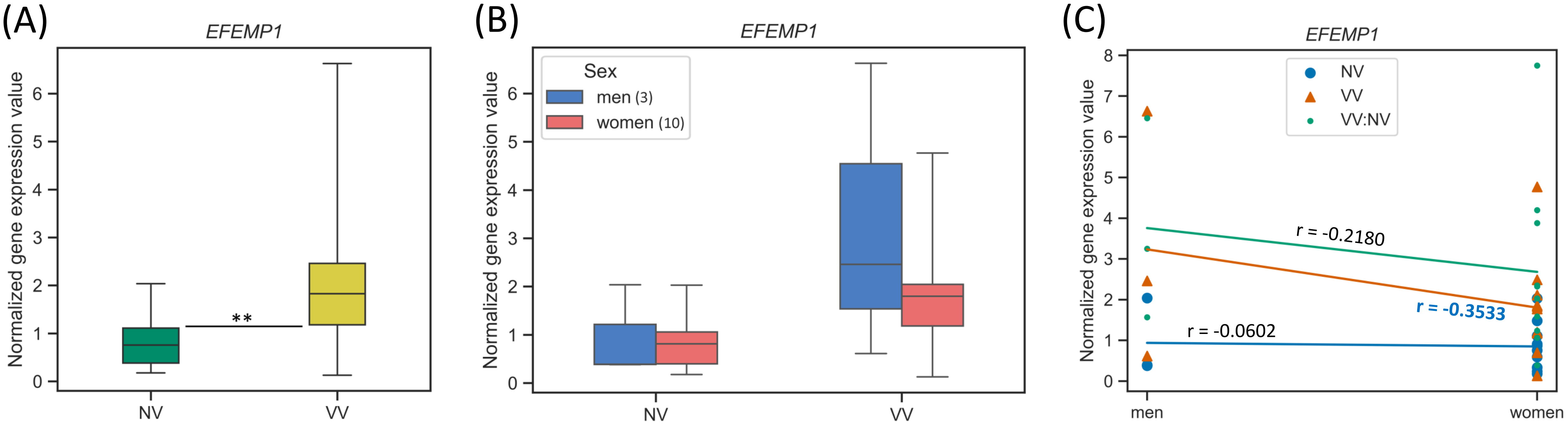

Figure S9. *EFEMP1* gene expression (mRNA level) data analysis. (**A**) Distribution of gene expression relative values in paired non-varicose vs. varicose vein segments in patients of the whole sample; (**B**) Distribution of gene expression relative values in non-varicose and varicose vein segments according to sex; (**C**) Scatter plot and the corresponding regression line for the relationship between the dependent (gene expression) variable and independent (sex) variable.

The box borders show the interquartile range, the horizontal line inside it indicates the median, and the whiskers show the maximum and minimum values; NV – non-varicose vein; VV – varicose vein; ** p-value < 0.01 (Wilcoxon test); r – correlation coefficient; r > |± 0.3| is displayed in blue; the number of patients of different sexes is indicated in the legend in the brackets.

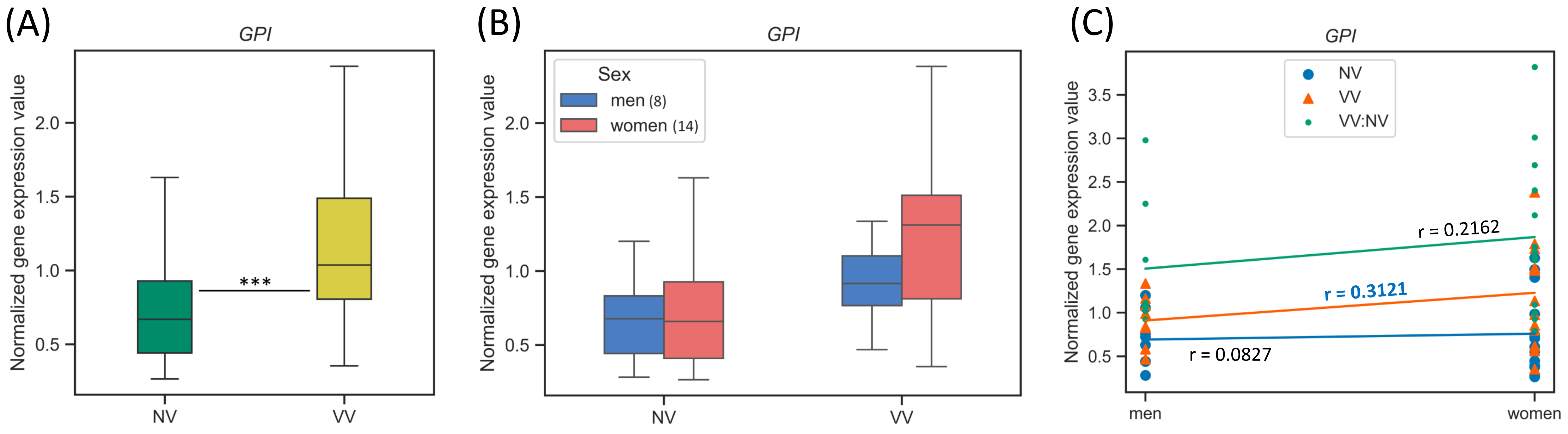

Figure S10. *GPI* gene expression (mRNA level) data analysis. (**A**) Distribution of gene expression relative values in paired non-varicose vs. varicose vein segments in patients of the whole sample; (**B**) Distribution of gene expression relative values in non-varicose and varicose vein segments according to sex; (**C**) Scatter plot and the corresponding regression line for the relationship between the dependent (gene expression) variable and independent (sex) variable.

The box borders show the interquartile range, the horizontal line inside it indicates the median, and the whiskers show the maximum and minimum values; NV – non-varicose vein; VV – varicose vein; *** p-value < 0.001 (Wilcoxon test); r – correlation coefficient; r > |± 0.3| is displayed in blue; the number of patients of different sexes is indicated in the legend in the brackets.

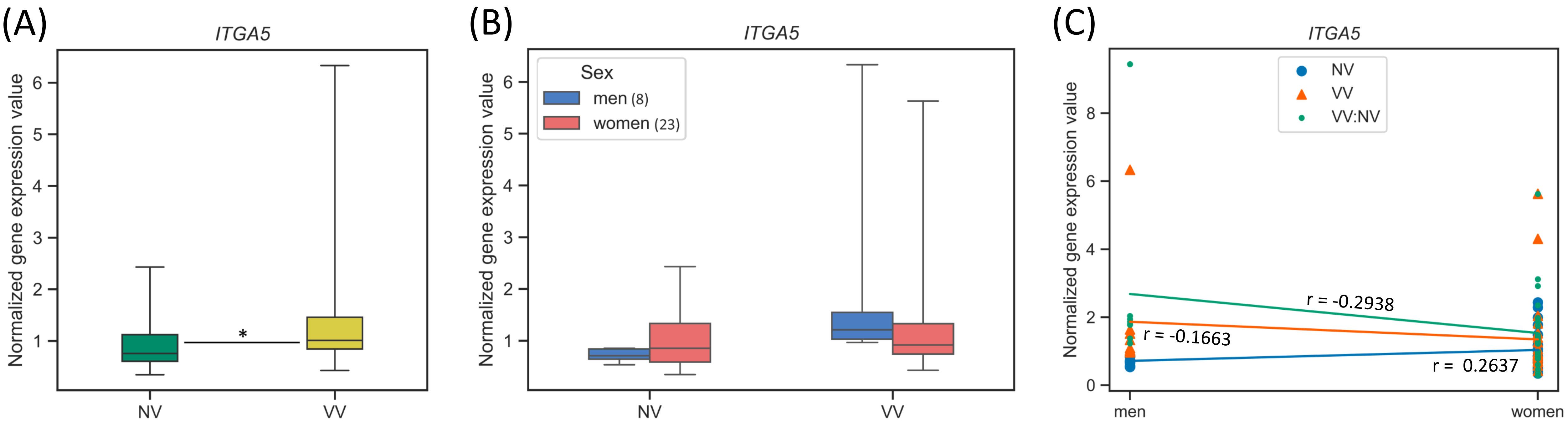

Figure S11. *ITGA5* gene expression (mRNA level) data analysis. (**A**) Distribution of gene expression relative values in paired non-varicose vs. varicose vein segments in patients of the whole sample; (**B**) Distribution of gene expression relative values in non-varicose and varicose vein segments according to sex; (**C**) Scatter plot and the corresponding regression line for the relationship between the dependent (gene expression) variable and independent (sex) variable.

The box borders show the interquartile range, the horizontal line inside it indicates the median, and the whiskers show the maximum and minimum values; NV – non-varicose vein; VV – varicose vein; * p-value < 0.05 (Wilcoxon test); r – correlation coefficient; the number of patients of different sexes is indicated in the legend in the brackets.

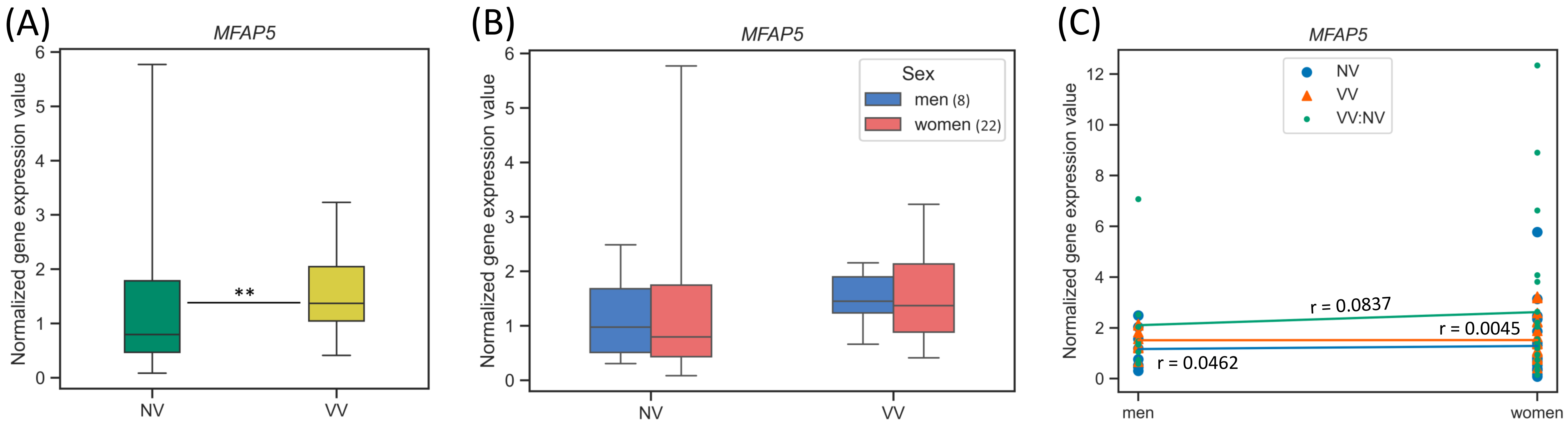

Figure S12. *MFAP5* gene expression (mRNA level) data analysis. (**A**) Distribution of gene expression relative values in paired non-varicose vs. varicose vein segments in patients of the whole sample; (**B**) Distribution of gene expression relative values in non-varicose and varicose vein segments according to sex; (**C**) Scatter plot and the corresponding regression line for the relationship between the dependent (gene expression) variable and independent (sex) variable.

The box borders show the interquartile range, the horizontal line inside it indicates the median, and the whiskers show the maximum and minimum values; NV – non-varicose vein; VV – varicose vein; ** p-value < 0.01 (Wilcoxon test); r – correlation coefficient; the number of patients of different sexes is indicated in the legend in the brackets.

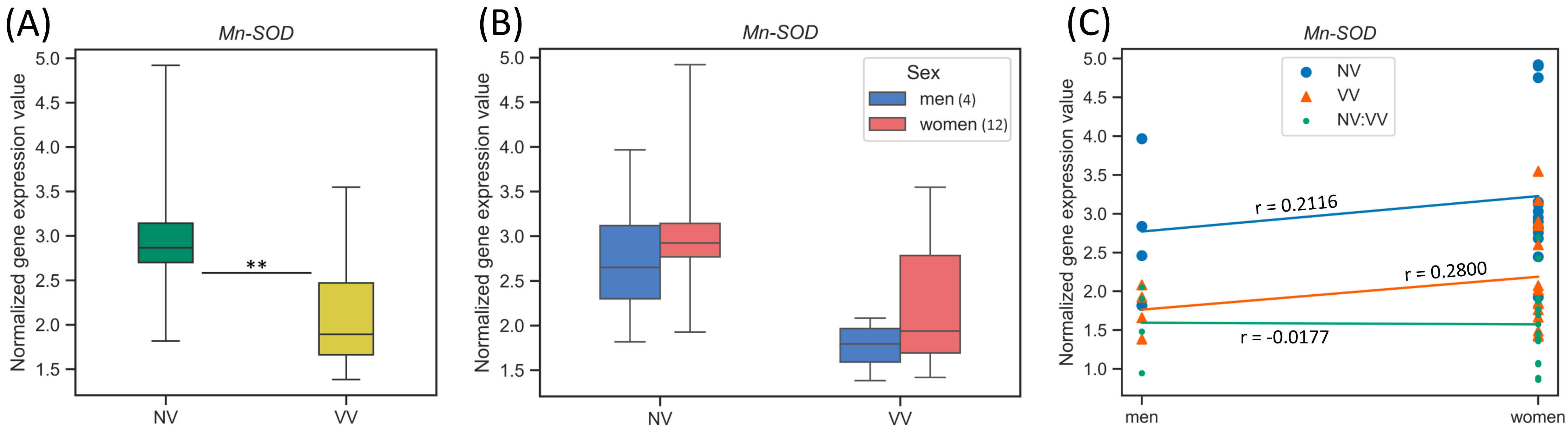

Figure S13. *Mn-SOD* gene expression (mRNA level) data analysis. (**A**) Distribution of gene expression relative values in paired non-varicose vs. varicose vein segments in patients of the whole sample; (**B**) Distribution of gene expression relative values in non-varicose and varicose vein segments according to sex; (**C**) Scatter plot and the corresponding regression line for the relationship between the dependent (gene expression) variable and independent (sex) variable.

The box borders show the interquartile range, the horizontal line inside it indicates the median, and the whiskers show the maximum and minimum values; NV – non-varicose vein; VV – varicose vein; ** p-value < 0.01 (Wilcoxon test); r – correlation coefficient; the number of patients of different sexes is indicated in the legend in the brackets.

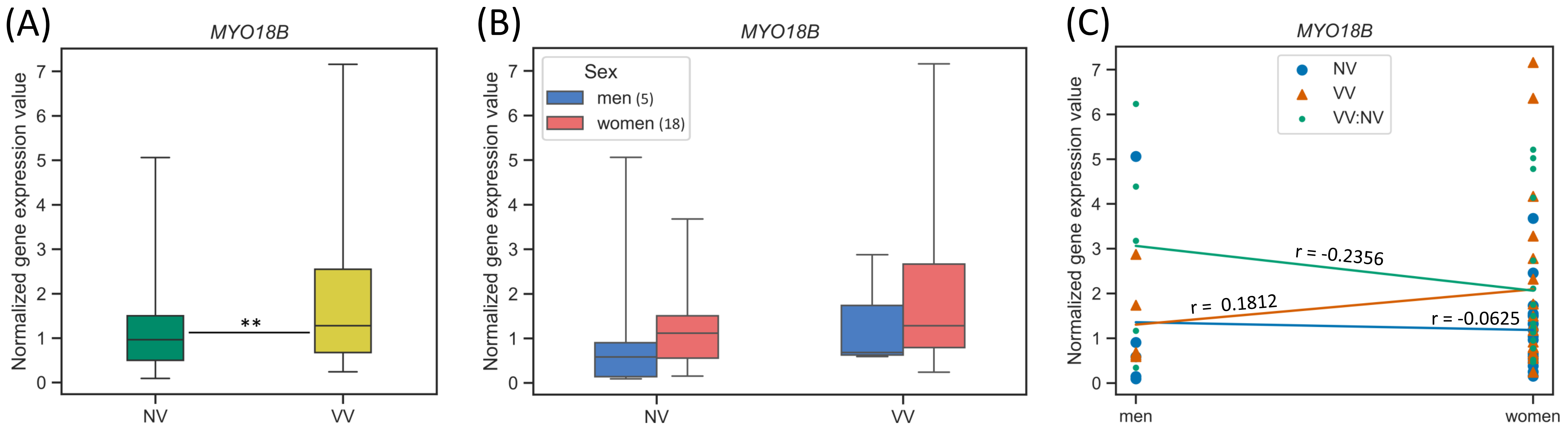

Figure S14. *MYO18B* gene expression (mRNA level) data analysis. (**A**) Distribution of gene expression relative values in paired non-varicose vs. varicose vein segments in patients of the whole sample; (**B**) Distribution of gene expression relative values in non-varicose and varicose vein segments according to sex; (**C**) Scatter plot and the corresponding regression line for the relationship between the dependent (gene expression) variable and independent (sex) variable.

The box borders show the interquartile range, the horizontal line inside it indicates the median, and the whiskers show the maximum and minimum values; NV – non-varicose vein; VV – varicose vein; ** p-value < 0.01 (Wilcoxon test); r – correlation coefficient; the number of patients of different sexes is indicated in the legend in the brackets.

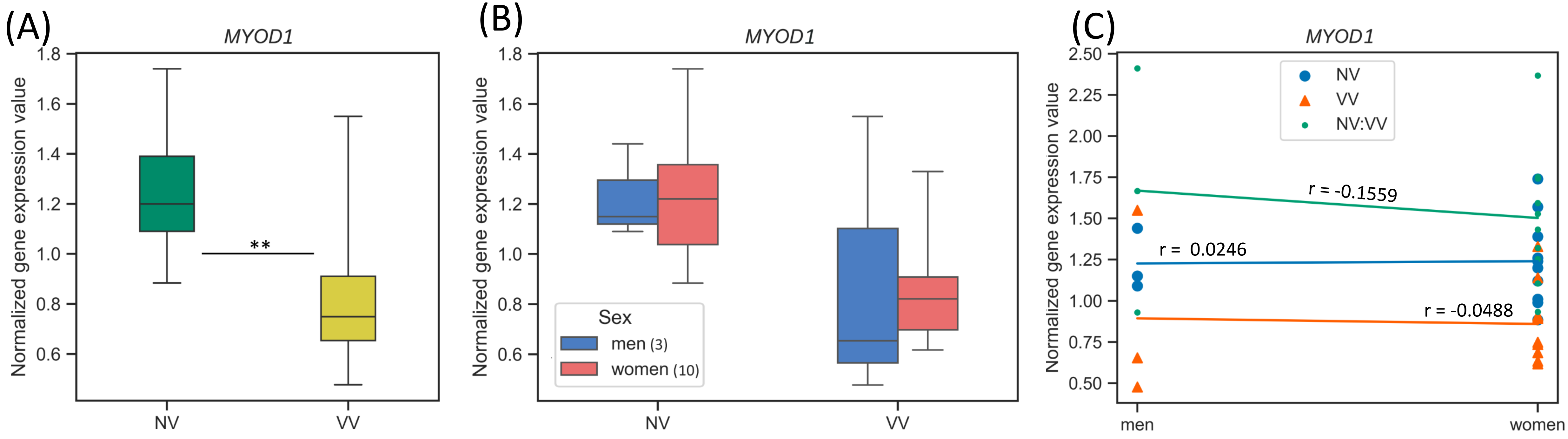

Figure S15. *MYOD1* gene expression (mRNA level) data analysis. (**A**) Distribution of gene expression relative values in paired non-varicose vs. varicose vein segments in patients of the whole sample; (**B**) Distribution of gene expression relative values in non-varicose and varicose vein segments according to sex; (**C**) Scatter plot and the corresponding regression line for the relationship between the dependent (gene expression) variable and independent (sex) variable.

The box borders show the interquartile range, the horizontal line inside it indicates the median, and the whiskers show the maximum and minimum values; NV – non-varicose vein; VV – varicose vein; ** p-value < 0.01 (Wilcoxon test); r – correlation coefficient; the number of patients of different sexes is indicated in the legend in the brackets.

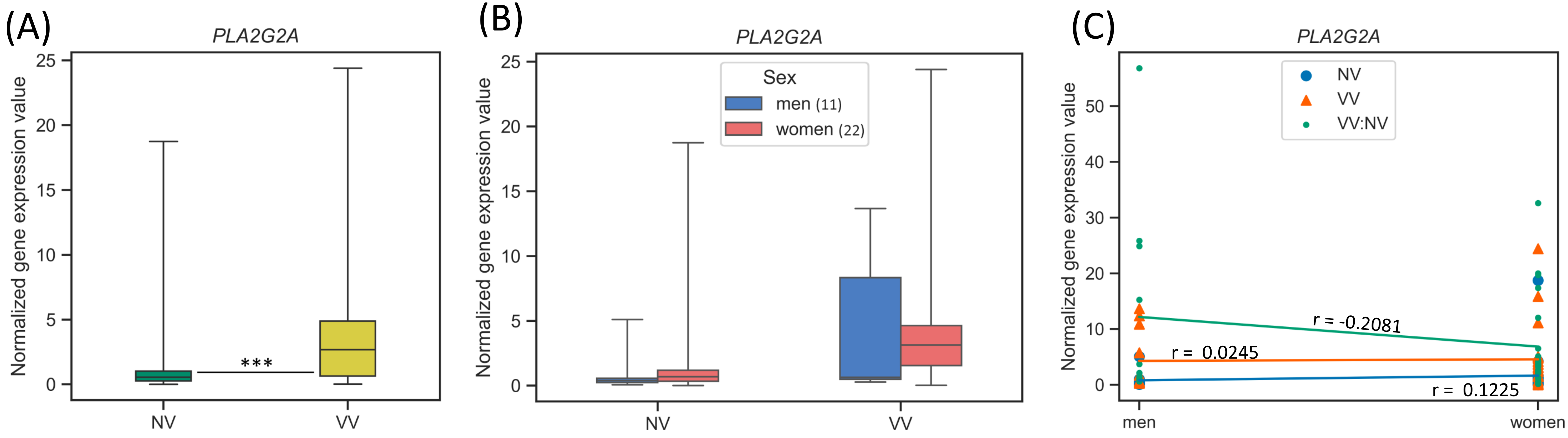

Figure S16. *PLA2G2A* gene expression (mRNA level) data analysis. (**A**) Distribution of gene expression relative values in paired non-varicose vs. varicose vein segments in patients of the whole sample; (**B**) Distribution of gene expression relative values in non-varicose and varicose vein segments according to sex; (**C**) Scatter plot and the corresponding regression line for the relationship between the dependent (gene expression) variable and independent (sex) variable.

The box borders show the interquartile range, the horizontal line inside it indicates the median, and the whiskers show the maximum and minimum values; NV – non-varicose vein; VV – varicose vein; *** p-value < 0.001 (Wilcoxon test); r – correlation coefficient; the number of patients of different sexes is indicated in the legend in the brackets.

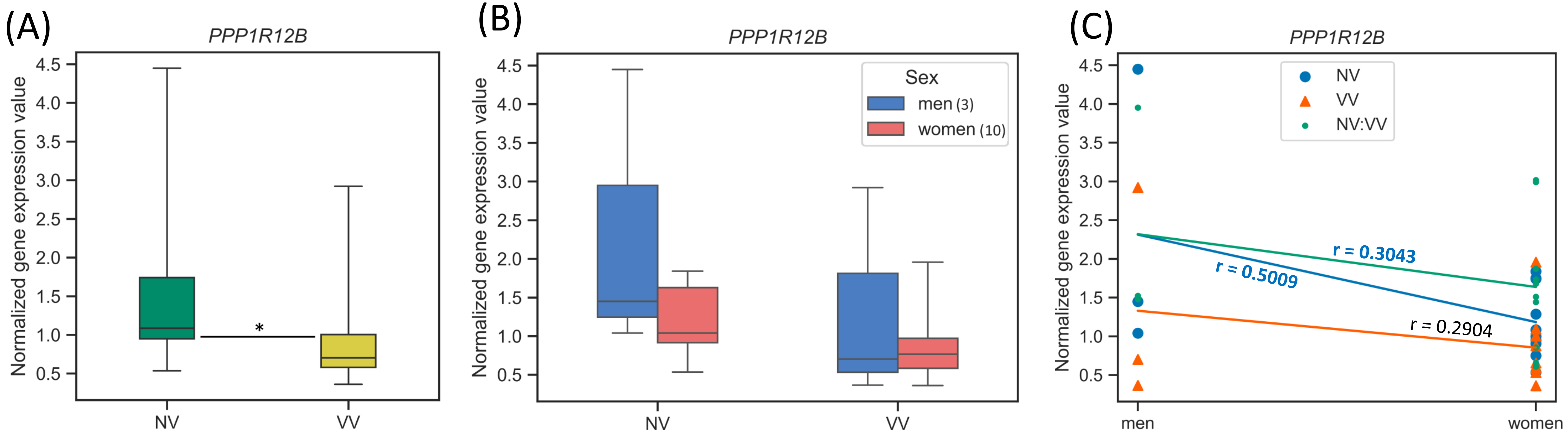

Figure S17. *PPP1R12B* gene expression (mRNA level) data analysis. (**A**) Distribution of gene expression relative values in paired non-varicose vs. varicose vein segments in patients of the whole sample; (**B**) Distribution of gene expression relative values in non-varicose and varicose vein segments according to sex; (**C**) Scatter plot and the corresponding regression line for the relationship between the dependent (gene expression) variable and independent (sex) variable.

The box borders show the interquartile range, the horizontal line inside it indicates the median, and the whiskers show the maximum and minimum values; NV – non-varicose vein; VV – varicose vein; * p-value < 0.05 (Wilcoxon test); r – correlation coefficient; r > |± 0.3| are displayed in blue; r > |± 0.5| is displayed in red color; the number of patients of different sexes is indicated in the legend in the brackets.

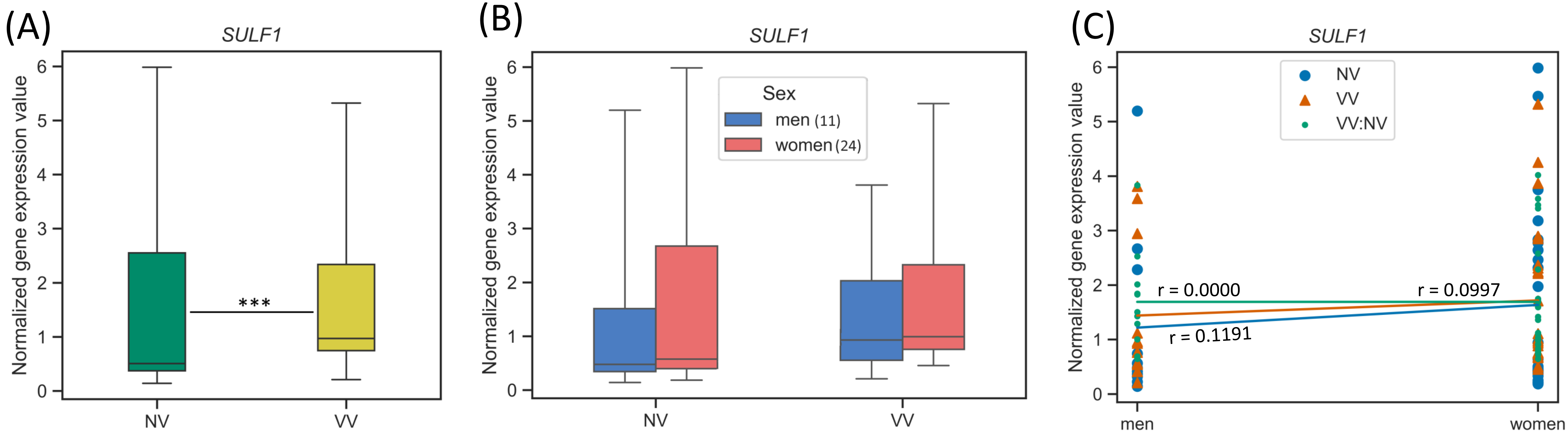

Figure S18. *SULF1* gene expression (mRNA level) data analysis. (**A**) Distribution of gene expression relative values in paired non-varicose vs. varicose vein segments in patients of the whole sample; (**B**) Distribution of gene expression relative values in non-varicose and varicose vein segments according to sex; (**C**) Scatter plot and the corresponding regression line for the relationship between the dependent (gene expression) variable and independent (sex) variable.

The box borders show the interquartile range, the horizontal line inside it indicates the median, and the whiskers show the maximum and minimum values; NV – non-varicose vein; VV – varicose vein; *** p-value < 0.001 (Wilcoxon test); r – correlation coefficient; the number of patients of different sexes is indicated in the legend in the brackets.

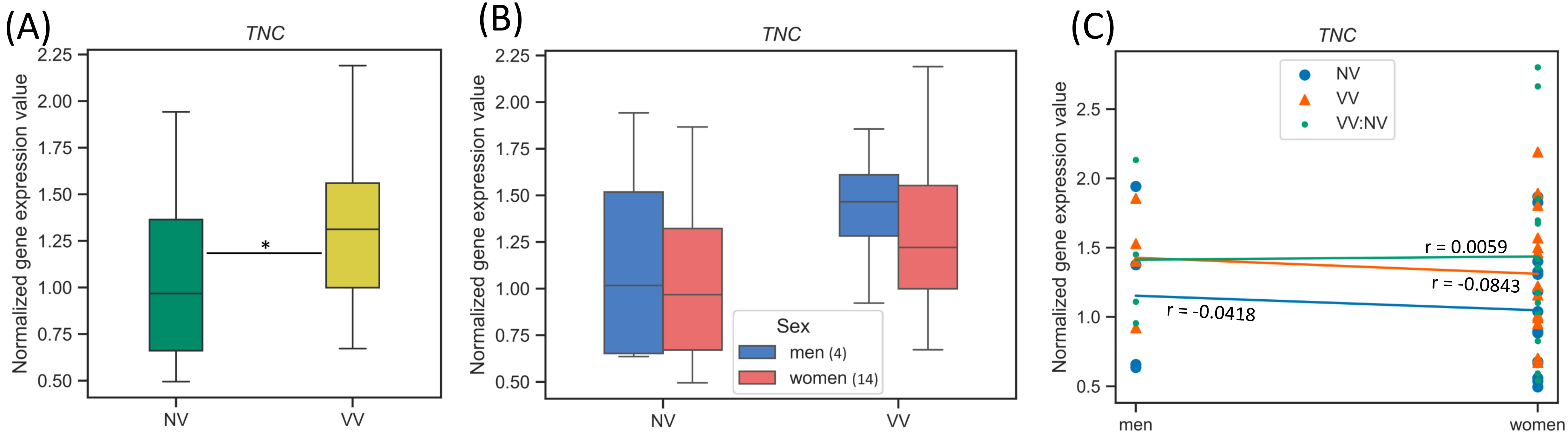

Figure S19. *TNC* gene expression (mRNA level) data analysis. (**A**) Distribution of gene expression relative values in paired non-varicose vs. varicose vein segments in patients of the whole sample; (**B**) Distribution of gene expression relative values in non-varicose and varicose vein segments according to sex; (**C**) Scatter plot and the corresponding regression line for the relationship between the dependent (gene expression) variable and independent (sex) variable.

The box borders show the interquartile range, the horizontal line inside it indicates the median, and the whiskers show the maximum and minimum values; NV – non-varicose vein; VV – varicose vein; * p-value < 0.05 (paired Student’s t-test); r – correlation coefficient; the number of patients of different sexes is indicated in the legend in the brackets.

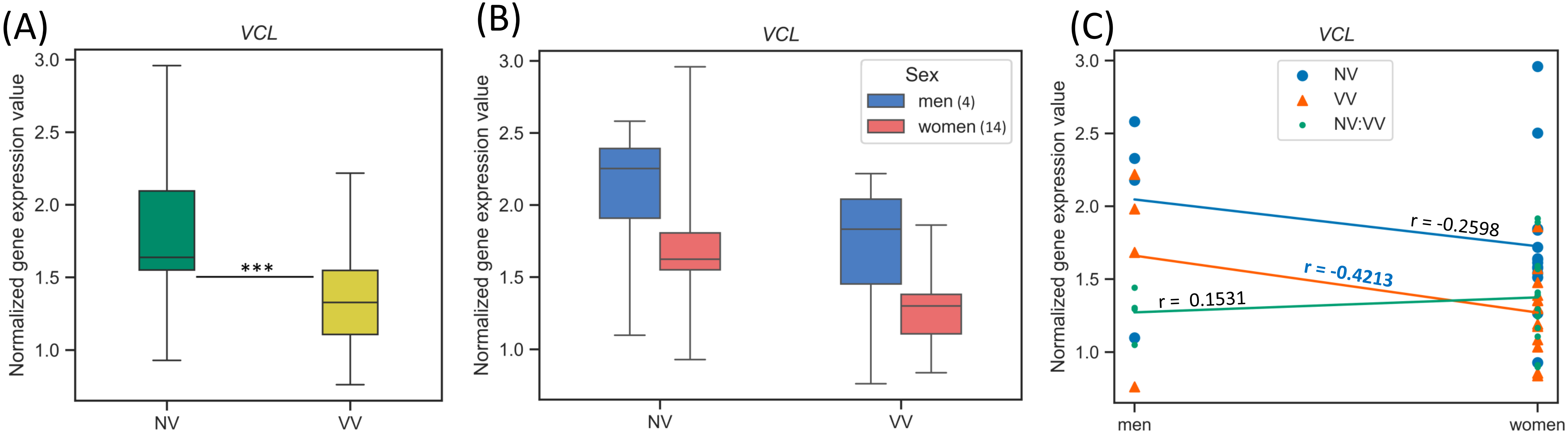

Figure S20. Analysis of data on the expression (mRNA level) of the *VCL* gene expression (mRNA level) data analysis. (**A**) Distribution of gene expression relative values in paired non-varicose vs. varicose vein segments in patients of the whole sample; (**B**) Distribution of gene expression relative values in non-varicose and varicose vein segments according to sex; (**C**) Scatter plot and the corresponding regression line for the relationship between the dependent (gene expression) variable and independent (sex) variable.

The box borders show the interquartile range, the horizontal line inside it indicates the median, and the whiskers show the maximum and minimum values; NV – non-varicose vein; VV – varicose vein; *** p-value < 0.001 (paired Student's t-test); r – correlation coefficient; r > |± 0.3| is displayed in blue; the number of patients of different sexes is indicated in the legend in the brackets.
