# Supplementary material for: Sex-Specific Gene Expression Differences in Varicose Veins
Source: Biomedicines. 2025 Sep 27;13(10):2373. doi: 10.3390/biomedicines13102373 (PMC12562233; doi:10.3390/biomedicines13102373)
Supplement: Supplementary file 1 [file biomedicines-13-02373-s001.zip › Supplementary Tables_revised.pdf]

Table S1. Primers used in the study.

Gene	Chr	Forward primer sequence	Reverse primer sequence
ABCA1	9	5′-AGCCAATCCTGAGAAC-3′	5′-TCCTGTCGCATGTCACTC-3′
AXL	19	5′-ACCTTCAACTCCTGCCTTCT-3′	5′-GCTTCTCCTTCAGCTCTTCA-3′
CALU	7	5′-ACGGCTACGTTTTAGATGATCC-3′	5′-TTGGTGGCAATGAGGTCTC-3′
CASZ1	1	5′-TCCACTGTCAAGAACGGACT-3′	5′-GCGGTACTGGTAGGCACAGT-3′
CCL2	17	5′-CTCAGCCAGATGCAATCAA-3′	5′-CACAATGGTCTTGAAGATCACA-3′
CHRDL2	11	5′-CAGTGTGCAGTCGCTCCAT-3′	5′-CATGGATCCTGAGGATGTCT-3′
COL15A1	9	5′-GGCACCAAGGAGGAACA-3′	5′-CCCTGGGAAGCAGTCTCT-3′
COMP	19	5′-GACAGTGATGGCGATGGTAT-3′	5′-GTCATTGTCGTCGTCGTCGT-3′
EBF1	5	5′-GGAGAAGGAAAAAGAAGCCAA-3′	5′-GCTCCGTCCTTATCCCATT-3′
EFEMP1	2	5′-CAGTGCGTAGACATAGATGA-3′	5′-TGATTGCTGGCATCACAT-3′
GPI	19	5′-ATTGCCTCCAAGACCTTTACTAC-3′	5′-TCACTTTGGTTGTGTTAGTAGACA-3′
ITGA5	12	5′-CCAAAAAGAGCGTCAGGT-3′	5′-GCTTGTAGAGGATGTAGATGA-3′
MFAP5	12	5′-TGCTCTCGTCTTGTCTGTAAGG-3′	5′-ACAGGGAGGAAGTCGGAAGT-3′
Mn-SOD	6	5′-AAAGGGGAGTTGCTGGAA-3′	5′-CCTTATTGAAACCAAGCCAA-3′
MYO18B	22	5′-GAAGAAGCAGAAGAAGTTTGACC-3′	5′-GTCACTTTCTCACGGAGACC-3′
MYOD1	11	5′-CGACGGCATGATGGACTACA-3′	5′-CGACACCGCCGCACTCTT-3′
PLA2G2A	1	5′-CAACGGATCGCTGCTG-3′	5′-AGCCTTATCACACTCACACA-3′
PPP1R12B	1	5′-TTAAGAACAAAGAGAAGATGCTC-3′	5′-GCTTTTTATCTGCCTCCTT-3′
STK38L	12	5′-CACCAAGTGACTTCTCATTTCAGA-3′	5′-ACTGTGGAATATGCCAGTTGTC-3′
SULF1	8	5′-GAAAATCTTCAAAGGACCCTAT-3′	5′-TCTCCGTCTCCTGATTTCTG-3′
TIMP1	X	5′-ATTCCGACCTCGTCATCA-3′	5′-GAACCCTTTATACATCTTGGTC-3′
TNC	9	5′-CACGTACTTACCTGCACCT-3′	5′-CTCCCTCATCTTCTTTATTCAT-3′
VCL	10	5′-GAGGCTGAGGTCCGTAAA-3′	5′-TGGCCATCTTAGTCATTCCT-3′
		Reference genes	
ACTB	7	5′-TGACCCAGATCATGTTTGAGA-3′	5′-CCAGAGGCGTACAGGGATA-3′
GAPDH	12	5′-CCTCAAGATCATCAGCAATG-3′	5′-GCATGGACTGTGGTCATG-3′

Table S2. Sex-related multiple linear regression analysis results of the expression of genes (listed in the alphabetical order) not included in figures of the main text (due to incomplete significance) and, for the reference (at the end of the Table), genes included in figures of the main text.

Gene [chromosome]	MLR model_ gene expression	Beta	p (Beta)	R	p (R)	GVIF* or VIF#
ABCA1 [chr 9]	*ABCA1*_NV vs. Sex*	0.025	0.925	**0.648**	**0.066**	1.138*
	*ABCA1*_VV vs. Sex*	-0.125	0.788	**0.796**	**0.063**	1.110*
	*ABCA1*_NV:VV ratio vs. Sex*	0.088	0.849	**0.490**	0.253	1.068*
	*ABCA1*_NV vs. Sex#	0.116	0.707	–	–	–
	*ABCA1*_VV vs. Sex#	-0.193	0.529	–	–	–
	*ABCA1*_NV:VV ratio vs. Sex#	**0.294**	0.330	**0.294**	0.330	1.095#
AXL [chr 19]	*AXL*_NV vs. Sex*	**0.398**	**0.094**	**0.888**	**0.002**	1.221*
	*AXL*_VV vs. Sex*	-0.069	0.872	**0.513**	**0.073**	1.147*
	*AXL*_NV:VV ratio vs. Sex*	**0.388**	0.392	**0.611**	0.219	1.068*
	*AXL*_NV vs. Sex#	**-0.435**	0.137	**0.435**	0.137	1.233#
	*AXL*_VV vs. Sex#	**-0.421**	0.152	**0.421**	0.152	1.215#
	*AXL*_NV:VV ratio vs. Sex#	-0.040	0.896	–	–	–
CALU [chr 7]	*CALU*_NV vs. Sex*	-0.147	0.304	**0.627**	**0.001**	1.078*
	*CALU*_VV vs. Sex*	-0.017	0.910	**0.556**	**0.003**	1.083*
	*CALU*_NV:VV ratio vs. Sex*	0.110	0.529	0.205	0.236	1.016*
	*CALU*_NV vs. Sex#	-0.169	0.333	–	–	–
	*CALU*_VV vs. Sex#	-0.059	0.737	–	–	–
	*CALU*_NV:VV ratio vs. Sex#	0.089	0.612	–	–	–
CASZ1 [chr 1]	*CASZ1*_NV vs. Sex*	**-0.330**	0.103	**0.596**	**0.044**	1.072*
	*CASZ1*_VV vs. Sex*	**0.282**	0.368	**0.548**	0.171	1.044*
	*CASZ1*_VV:NV ratio vs. Sex*	**0.598**	**0.034**	**0.696**	**0.018**	1.083*
	*CASZ1*_NV vs. Sex#	-0.246	0.269	0.246	0.269	1,065#
	*CASZ1*_VV vs. Sex#	-0.066	0.771	–	–	–
	*CASZ1*_VV:NV ratio vs. Sex#	0.161	0.475	–	–	–
CCL2 [chr 17]	*CCL2*_NV vs. Sex*	0.067	0.755	**0.545**	**0.013**	1.055*
	*CCL2*_VV vs. Sex*	0.027	0.906	**0.418**	**0.051**	1.042*
	*CCL2*_ NV:VV ratio vs. Sex*	0.022	0.898	**0.262**	0.135	1.027*
	*CCL2*_NV vs. Sex#	0.173	0.328	–	–	–
	*CCL2*_VV vs. Sex#	-0.120	0.498	–	–	–
	*CCL2*_ NV:VV ratio vs. Sex#	0.002	0.989	–	–	–
CHRDL2 [chr 11]	*CHRDL2*_NV vs. Sex*	**-0.274**	0.363	**0.327**	0.275	1.052*
	*CHRDL2*_VV vs. Sex*	**-0.470**	0.105	**0.470**	0.105	1.119*
	*CHRDL2*_ VV:NV ratio vs. Sex*	0.207	0.417	**0.652**	**0.063**	1.141*
	*CHRDL2*_NV vs. Sex#	-0.308	0.305	0.308	0.305	1.105#
	*CHRDL2*_VV vs. Sex#	-0.470	0.105	0.470	0.105	1.284#
	*CHRDL2*_ VV:NV ratio vs. Sex#	0.197	0.520	–	–	–

Table S2 (continued)

Gene [chromosome]	MLR model_ gene expression	Beta	p (Beta)	R	p (R)	GVIF* or VIF#
COL15A1 [chr 9]	*COL15A1*_NV vs. Sex*	-0.098	0.740	0.610	0.098	1.117*
	*COL15A1*_VV vs. Sex*	-0.094	0.812	0.831	0.011	1.163*
	*COL15A1*_ VV:NV ratio vs. Sex*	0.302	0.533	0.702	0.093	1.091*
	*COL15A1*_NV vs. Sex#	0.140	0.648	–	–	–
	*COL15A1*_VV vs. Sex#	-0.293	0.331	0.293	0.331	1.094#
	*COL15A1*_ VV:NV ratio vs. Sex#	-0.433	0.139	0.433	0.139	1.231#
COMP [chr 19]	*COMP*_NV vs. Sex*	-0.328	0.136	0.717	0.015	1.122*
	*COMP*_VV vs. Sex*	-0.174	0.410	0.592	0.010	1.204*
	*COMP*_VV:NV ratio vs. Sex*	-0.170	0.337	0.789	0.001	1.254*
	*COMP*_NV vs. Sex#	-0.004	0.987	–	–	–
	*COMP*_VV vs. Sex#	-0.083	0.742	–	–	–
	*COMP*_VV:NV ratio vs. Sex#	-0.234	0.350	–	–	–
EFEMP1 [chr 2]	*EFEMP1*_NV vs. Sex*	0.202	0.679	0.768	0.095	1.097*
	*EFEMP1*_VV vs. Sex*	0.152	0.735	0.573	0.137	1.099*
	*EFEMP1*_ VV:NV ratio vs. Sex*	0.021	0.906	0.865	0.001	1.390*
	*EFEMP1*_NV vs. Sex#	-0.060	0.845	–	–	–
	*EFEMP1*_VV vs. Sex#	-0.353	0.236	0.353	0.236	1.143#
	*EFEMP1*_ VV:NV ratio vs. Sex#	-0.218	0.474	–	–	–
GPI [chr 19]	*GPI*_NV vs. Sex*	0.061	0.736	0.624	0.002	1.229*
	*GPI*_VV vs. Sex*	0.151	0.588	0.539	0.039	1.081*
	*GPI*_ VV:NV ratio vs. Sex*	0.227	0.298	0.381	0.226	1.036*
	*GPI*_NV vs. Sex#	0.083	0.714	–	–	–
	*GPI*_VV vs. Sex#	0.312	0.157	0.312	0.157	1.108#
	*GPI*_ VV:NV ratio vs. Sex#	0.216	0.334	–	–	–
ITGA5 [chr 12]	*ITGA5*_NV vs. Sex*	0.371	0.051	0.448	0.115	1.035*
	*ITGA5*_VV vs. Sex*	0.016	0.937	0.471	0.085	1.039*
	*ITGA5*_VV:NV ratio vs. Sex*	-0.103	0.597	0.518	0.041	1.049*
	*ITGA5*_NV vs. Sex#	0.264	0.152	0.264	0.152	1.075#
	*ITGA5*_VV vs. Sex#	-0.166	0.371	–	–	–
	*ITGA5*_VV:NV ratio vs. Sex#	-0.294	0.109	0.294	0.109	1.094#
MFAP5 [chr 12]	*MFAP5*_NV vs. Sex*	-0.109	0.563	0.646	0.005	1.087*
	*MFAP5*_VV vs. Sex*	-0.423	0.051	0.619	0.026	1.059*
	*MFAP5*_VV:NV ratio vs. Sex*	0.046	0.787	0.667	0.003	1.095*
	*MFAP5*_NV vs. Sex#	0.046	0.808	–	–	–
	*MFAP5*_VV vs. Sex#	0.005	0.981	–	–	–
	*MFAP5*_VV:NV ratio vs. Sex#	0.084	0.660	–	–	–

Table S2 (continued)

Gene [chromosome]	MLR model_ gene expression	Beta	p (Beta)	R	p (R)	GVIF* or VIF#
Mn-SOD [chr 6]	*Mn-SOD*_NV vs. Sex*	-0.009	0.972	**0.507**	**0.045**	1.139*
	*Mn-SOD*_VV vs. Sex*	**0.390**	0.189	**0.378**	0.367	1.037*
	*Mn-SOD*_ NV:VV ratio vs. Sex*	-0.096	0.724	–	–	–
	*Mn-SOD*_NV vs. Sex#	0.212	0.399	–	–	–
	*Mn-SOD*_VV vs. Sex#	**0.280**	0.260	**0.280**	0.260	1.085#
	*Mn-SOD*_ NV:VV ratio vs. Sex#	-0.017	0.944	–	–	–
MYO18B [chr 22]	*MYO18B*_NV vs. Sex*	-0.062	0.777	–	–	–
	*MYO18B*_VV vs. Sex*	0.181	0.408	–	–	–
	*MYO18B*_VV:NV ratio vs. Sex*	0.015	0.968	**0.300**	0.165	1.040*
	*MYO18B*_NV vs. Sex#	-0.062	0.777	–	–	–
	*MYO18B*_VV vs. Sex#	0.181	0.408	–	–	–
	*MYO18B*_VV:NV ratio vs. Sex#	-0.236	0.279	0.236	0.279	1.059#
MYOD1 [chr 11]	*MYOD1*_NV vs. Sex*	-0.128	0.636	**0.728**	**0.067**	1.102*
	*MYOD1*_VV vs. Sex*	**-0.470**	0.105	**0.470**	0.105	1.061*
	*MYOD1*_ NV:VV ratio vs. Sex*	0.207	0.417	**0.652**	**0.063**	1.141*
	*MYOD1*_NV vs. Sex#	0.025	0.937	–	–	–
	*MYOD1*_VV vs. Sex#	-0.049	0.874	–	–	–
	*MYOD1*_ NV:VV ratio vs. Sex#	-0.156	0.611	–	–	–
PLA2G2A [chr 1]	*PLA2G2A*_NV vs. Sex*	0.128	0.475	0.195	0.277	1.015*
	*PLA2G2A*_VV vs. Sex*	0.030	0.865	**0.319**	0.201	1.024*
	*PLA2G2A*_ VV:NV ratio vs. Sex*	0.148	0.524	**0.424**	**0.051**	1.044*
	*PLA2G2A*_NV vs. Sex#	0.122	0.497	–	–	–
	*PLA2G2A*_VV vs. Sex#	0.025	0.892	–	–	–
	*PLA2G2A*_ VV:NV ratio vs. Sex#	-0.208	0.245	0.208	0.245	1.045#
PPP1R12B [chr 1]	*PPP1R12B*_NV vs. Sex*	**0.517**	**0.061**	**0.631**	**0.079**	1.128*
	*PPP1R12B*_VV vs. Sex*	**0.290**	0.336	**0.290**	0.336	1.040*
	*PPP1R12B*_NV:VV ratio vs. Sex*	-0.132	0.780	**0.326**	0.277	1.051*
	*PPP1R12B*_NV vs. Sex#	**-0.501**	**0.081**	**0.501**	**0.081**	1.335#
	*PPP1R12B*_VV vs. Sex#	**-0.290**	0.336	**0.290**	0.336	1.092#
	*PPP1R12B*_ NV:VV ratio vs. Sex#	**-0.304**	0.312	**0.304**	0.312	1.102#
SULF1 [chr 8]	*SULF1*_NV vs. Sex*	0.043	0.780	**0.578**	**0.013**	1.048*
	*SULF1*_VV vs. Sex*	0.028	0.856	**0.548**	**0.010**	1.056*
	*SULF1*_ VV:NV ratio vs. Sex*	0.015	0.933	0.210	0.226	1.017*
	*SULF1*_NV vs. Sex#	0.119	0.495	–	–	–
	*SULF1*_VV vs. Sex#	0.1	0.569	–	–	–
	*SULF1*_ VV:NV ratio vs. Sex#	0.000	1	–	–	–

Table S2 (continued)

Gene [chromosome]	MLR model_ gene expression	Beta	p (Beta)	R	p (R)	GVIF* or VIF#
TNC [chr 9]	*TNC*_NV vs. Sex*	-0.042	0.878	–	–	–
	*TNC*_VV vs. Sex*	0.154	0.645	0.570	0.179	1.065*
	*TNC*_VV:NV ratio vs. Sex*	0.002	0.995	0.479	0.183	1.063*
	*TNC*_NV vs. Sex#	-0.094	0.712	–	–	–
	*TNC*_VV vs. Sex#	-0.116	0.646	–	–	–
	*TNC*_VV:NV ratio vs. Sex#	0.016	0.949	–	–	–
VCL [chr 10]	*VCL*_NV vs. Sex*	0.038	0.863	0.799	0.005	1.177*
	*VCL*_VV vs. Sex*	0.208	0.351	0.839	0.002	1.215*
	*VCL*_NV:VV ratio vs. Sex*	-0.243	0.351	0.798	0.017	1.131*
	*VCL*_NV vs. Sex#	-0.260	0.298	0.260	0.298	1.072#
	*VCL*_VV vs. Sex#	-0.421	0.082	0.421	0.082	1.216#
	*VCL*_NV:VV ratio vs. Sex#	0.153	0.544	–	–	–
STK38L [chr 12]	*STK38L*_NV vs. Sex*	0.287	0.152	0.440	0.094	1.050*
	*STK38L*_VV vs. Sex*	0.504	0.013	0.516	0.033	1.072*
	*STK38L*_VV:NV ratio vs. Sex*	-0.058	0.775	0.441	0.092	1.050*
	*STK38L*_NV vs. Sex#	0.326	0.112	0.326	0.112	1.119#
	*STK38L*_VV vs. Sex#	0.449	0.024	0.449	0.024	1.252#
	*STK38L*_VV:NV ratio vs. Sex#	-0.153	0.466	–	–	–
TIMP1 [chr X]	*TIMP1*_NV vs. Sex*	-0.472	0.097	0.712	0.083	1.095*
	*TIMP1*_VV vs. Sex*	-0.155	0.594	0.867	0.004	1.196*
	*TIMP1*_VV:NV ratio vs. Sex*	0.113	0.643	0.957	0.001	1.243*
	*TIMP1*_NV vs. Sex#	-0.191	0.533	–	–	–
	*TIMP1*_VV vs. Sex#	-0.612	0.026	0.612	0.026	1.598#
	*TIMP1*_VV:NV ratio vs. Sex#	-0.513	0.073	0.513	0.073	1.356#
EBF1 [chr 5]	*EBF1*_NV vs. Sex*	-0.659	0.027	0.642	0.070	1.135*
	*EBF1*_VV vs. Sex*	-1.609	0.002	0.895	0.021	1.152*
	*EBF1*_NV:VV ratio vs. Sex*	0.456	0.347	0.794	0.145	1.091*
	*EBF1*_NV vs. Sex#	-0.560	0.046	0.560	0.046	1.458#
	*EBF1*_VV vs. Sex#	-0.471	0.104	0.471	0.104	1.286#
	*EBF1*_NV:VV ratio vs. Sex#	-0.243	0.424	–	–	–

NV – non-varicose vein; VV – varicose vein; MLR - multiple linear regression; Beta – regression coefficient; R – coefficient of multiple correlation (the positive square root of the coefficient of multiple determination); VIF – variance inflation factor determined by the equation: $VIF=1/(1-R^2)$; GVIF – generalized variance inflation factor determined by the equation: GVIF=VIF^(1/(2*df)) where df is a degree of freedom; * all parameters (independent predictor variables) applied together: Sex, Age, BMI, VVD manifestation, CEAP class, Height, VVD duration (data is provided only for Sex); # Sex is the only independent predictor variable; Beta and R > ǀ± 0.25ǀ are displayed in blue color at p-value > 0.05; Beta and R > ǀ± 0.25ǀ are displayed in red color at p-value < 0.05; p-values < 0.05 are displayed in red color; 0.05 < p-values < 0.1 are displayed in blue color. Empty graphs ("-") mean that MLR analysis results are not available (there were no variables in the regression equation).

Table S3. Sex hormone-related TFBS within regulatory regions of the genes (listed in the alphabetical order) not included in Table 2 of the main text.

Gene	Chromosome	Number of TFBS[†]: total (in the promoter; in the enhancer(s))	Affinity score[‡]	Affinity p-value[§]
ABCA1	9	156 (9; 147) for AR and PR	12.999	1.21×10^{-5}
		53 (0; 53) for ER-alpha, ER-beta, ERR1, ERR2, ERR3	8.67	2.24×10^{-4}
AXL	19	4 (2; 2) for AR and PR	7.83	9.25×10^{-3}
		4 (1; 3) for ER-alpha, ER-beta, ERR1, ERR2, ERR3	7.68	8.94×10^{-3}
CALU	7	17 (0; 17) for AR and PR	4.87	1.79×10^{-5}
		36 (0; 36) for ER-alpha, ER-beta, ERR1, ERR2, ERR3	10.5	2.43×10^{-4}
CASZ1	1	47 (0; 47) for AR and PR	12.99	9.4×10^{-6}
		32 (0; 32) for ER-alpha, ER-beta, ERR1, ERR2, ERR3	6.11	2.52×10^{-3}
CCL2	17	31 (0; 31) for AR and PR	9.81	4.19×10^{-3}
		38 (6; 32) for ER-alpha, ER-beta, ERR1, ERR2, ERR3	8.65	7.34×10^{-4}
CHRDL2	11	30 (2; 28) for AR and PR	11.16	7.52×10^{-5}
		12 (2; 10) for ER-alpha, ER-beta, ERR1, ERR2, ERR3	6.49	5.37×10^{-3}
COL15A1	9	20 (0; 20) for AR and PR	6.69	1.25×10^{-2}
		17 (3; 14) for ER-alpha, ER-beta, ERR1, ERR2, ERR3	10.59	2.4×10^{-4}
COMP	19	2 (1; 1) for AR and PR	-10.84	2.06×10^{-2}
		4 (0; 4) for ER-alpha, ER-beta, ERR1, ERR2, ERR3	7.38	3.78×10^{-3}
EFEMP1	2	77 (5; 72) for AR and PR	12.4	4.86×10^{-5}
		31 (3; 28) for ER-alpha, ER-beta, ERR1, ERR2, ERR3	6.65	1.57×10^{-2}
GPI	19	11 (2; 9) for AR and PR	8.08	2.44×10^{-2}
		7 (0; 7) for ER-alpha, ER-beta, ERR1, ERR2, ERR3	8.67	3.63×10^{-3}
MFAP5	12	1 (0; 1) for AR and PR	7.58	1.34×10^{-2}
		2 (2; 0) for ER-alpha, ER-beta, ERR1, ERR2, ERR3	6.45	1.7×10^{-2}
Mn-SOD	6	–	–	–
		–	–	–
MYO18B	22	38 (1; 37) for AR and PR	9.51	6.16×10^{-3}
		21 (0; 21) for ER-alpha, ER-beta, ERR1, ERR2, ERR3	8.39	5.19×10^{-3}
MYOD1	11	26 (0; 26) for AR and PR	9.81	4.22×10^{-3}
		5 (0; 5) for ER-alpha, ER-beta, ERR1, ERR2, ERR3	10.2	2.25×10^{-3}
PLA2G2A	1	15 (4; 11) for AR and PR	6.70	3.41×10^{-2}
		6 (0; 6) for ER-alpha, ER-beta, ERR1, ERR2, ERR3	7.78	4.4×10^{-3}
SULF1	8	21 (2; 19) for AR and PR	5.41	1×10^{-100}
		19 (2; 17) for ER-alpha, ER-beta, ERR1, ERR2, ERR3	6.75	1.98×10^{-2}
TNC	9	108 (0; 108) for AR and PR	9.24	5.84×10^{-3}
		26 (1; 25) for ER-alpha, ER-beta, ERR1, ERR2, ERR3	11.17	7.64×10^{-4}

[†] TFBS: transcription factor binding sites; transcription factors (sex hormone receptors) associated with the respective matrices: AR – androgen receptor; PR – progesterone receptor; ER-alpha – estrogen receptor 1; ER-beta – estrogen receptor 2; ERR1 – estrogen related receptor alpha; ERR2 – estrogen related receptor beta; ERR3 – estrogen related receptor gamma.

[‡] Affinity score of the matrix estimates the overall affinity of a binding specificity to each of the studied regulatory regions of the respective gene; it corresponds to its best (minimum) affinity p-value among studied regulatory regions.

[§] Affinity p-value: the statistical significance of affinity scores estimated for large sets of random sequences with the dinucleotide composition of human promoters and sequence lengths ranging from 100 to 2500; it is taken from the best matrix (the most relevant and determining the regulation of the analyzed gene) corresponding to the given factors and represents the best affinity p-value among all examined regulatory regions of the respective gene.
